# The efficacy of Jianpi Yiqi therapy for chronic atrophic gastritis: A systematic review and meta-analysis

**DOI:** 10.1371/journal.pone.0181906

**Published:** 2017-07-24

**Authors:** Yun-kai Dai, Yun-zhan Zhang, Dan-yan Li, Jin-tong Ye, Ling-feng Zeng, Qi Wang, Ling Hu

**Affiliations:** 1 Institute of Gastroenterology, Guangzhou University of Chinese Medicine, Guangzhou, Guangdong, China; 2 Institute of Clinical Pharmacology, Guangzhou University of Chinese Medicine, Guangzhou, Guangdong, China; China Medical University, TAIWAN

## Abstract

Jianpi Yiqi therapy (JYT) is a classical therapy in treating chronic atrophic gastritis (CAG), but the clinical effects of it are still contentious. The purpose of this article is to evaluate the efficacy and safety of JYT for CAG. Seven electronic databases including PubMed, Embase, Springer Link, CNKI (China National Knowledge Infrastructure), VIP (Chinese Scientific Journals Database), Wan-fang database, and CBM (Chinese Biomedicine Database) were searched from their inception to November 1, 2016. 13 randomized controlled trials (RCTs) with a total of 1119 participants were identified for analysis. Meta-analyses demonstrated that both JYT (RR 1.41; 95% CI 1.27, 1.57; *P* < 0.00001) and JYT + western medicine (RR 1.27; 95% CI 1.17, 1.38; *P* < 0.00001) were more efficacious than only western medicine. Furthermore, JYT had potential improvement on traditional Chinese medicine (TCM) symptoms scores such as stomachache, stomach distention, belching, fatigue, et al. In addition, no serious adverse events were reported in the selected trials. The Cochrane Collaboration’s risk of bias tool was evaluated for the weaknesses of methodological quality, while the quality level of Grades of Recommendations Assessment Development and Evaluation (GRADE) evidence classification indicated “Very low”. This meta-analysis indicates that JYT may have potential effects on the treatment of patients with CAG. However, due to limitations of methodological quality and small sample size of the included studies, further standardized research of rigorous design should be needed.

## Introduction

Chronic atrophic gastritis (CAG), characterized by a loss of normal glandular structures and a collapse of the reticular skeleton of the gastric mucosa (GM), is a well-established premalignant lesion of gastric cancer (GC) in the absence of specific clinical manifestations [1–3]. Recently, with the increasing of incidence and prevalence of CAG in China, the risk of GC has been growing, causing significant reductions in patients’ quality of life and increasing substantial healthcare costs [4].

Although great progress has been made in elaborating the pathogenesis of CAG, most western medicines, including *Helicobacter pylori* (Hp) eradication, acid suppression, and nonsteroidal anti-inflammatory drugs, remain unsatisfied [5]. Due to chronicity and recurrence of this disease, many sufferers have put their concentrations on alternative treatments such as traditional Chinese medicine (TCM). Invigorating spleen and reinforcing qi (Chinese name in pinyin “Jianpi Yiqi”) is a classical Chinese therapy in treating CAG [6]. However, the current state of evidence of Jianpi Yiqi therapy (JYT) for CAG has been unknown. Therefore, a systematic review and meta-analysis of randomized, conventional western medicine controlled trials was conducted to evaluate its efficacy and safety.

## Materials and methods

### Search strategy

We comprehensively searched for publications in the following electronic databases from their inception through November 1, 2016: PubMed, Embase, Springer Link, CNKI (China National Knowledge Infrastructure), VIP (Chinese Scientific Journals Database), Wan-fang database, CBM (Chinese Biomedicine Database). The following general wording of the search terms were individually used or in combination: “traditional Chinese medicine”, “Chinese herbal medicine”, “herbal formula”, “herbs”, “alternative medicine”, “Jianpi”, “Yiqi”, “chronic atrophic gastritis”, “atrophic gastritis”, “precancerous lesions of gastric cancer”, “randomized controlled trial”. No restriction for publication was placed on language. Electronic searches of omissive relevant studies were supplemented by the manual searches. As for the grey literature, we had retrieved them through trying our best to contact with manufacturers and pharmacists who produced herbal formulae based on Jianpi Yiqi Therapy.

### Selection criteria

Studies meeting all of the following criteria were conducted in this meta-analysis. (1) Patients were definitely diagnosed CAG by gastroscopy and pathology. (2) The age of all participants was above 18 years old. (3) Experiment groups used Chinese herbal medicine of JYT, while control groups used conventional western medicine. (4) Treatment course was not less than 1 month. (5) The Jadad score was not less than 1.

### Data abstraction and quality assessment

Two researchers independently conducted data extraction, including first author, publication year, whether by Hp infection, type of syndrome, sex, sample size, age, course of disease, intervention, duration, outcome measures, follow-up and side effects. We used the Cochrane Collaboration’s risk of bias tool, conducted by Jadad scale in preliminary, to make evaluation of methodological quality. We could judge quality of literature from randomization, double blinding, and withdrawal or dropout in preliminary. The guidelines for assessment about Jadad scale are as follows:

1. Randomization: A method to generate the sequence of randomization will be regarded as appropriate if it allowed each study participant to have the same chance of receiving each intervention and the investigators could not predict which treatment was next. Methods of allocation using date of birth, date of admission, hospital numbers, or alternation should be not regarded as appropriate.
2. Double blinding: A study must be regarded as double blind if the word “double blind” is used. The method will be regarded as appropriate if it is stated that neither the person doing the assessments nor the study participant could identify the intervention being assessed, or if in the absence of such a statement the use of active placebos, identical placebos, or dummies is mentioned.
3. Withdrawals and dropouts: Participants who were included in the study but did not complete the observation period or who were not included in the analysis must be described. The number and the reasons for withdrawal in each group must be stated. If there were no withdrawals, it should be stated in the article. If there is no statement on withdrawals, this item must be given no points.

However, the final results of literature quality including the risk of bias evaluation were illustrated by the Cochrane tool. Disagreements were resolved after consulting with a third investigator.

### Data synthesis and analysis

Review Manager 5.3 software was used for this statistical analysis. We calculated the pooled risk ratio (RR) to assess dichotomous data, while the standardized mean difference (SMD) or mean difference (MD) was used for continuous variable data, with both adopting 95% confidence intervals (CI). Heterogeneity was statistically assessed by using the *χ*^*2*^ test and inconsistency index statistic (*I*^*2*^) [7]. A model of random effect was conducted if substantial heterogeneity existed (*I*^*2*^ >50% or *P*<0.05). We investigated possible sources of substantial heterogeneity using sensitivity analysis, which aimed at evaluating the robustness of emerging results through omitting one trial in turn. The number needed to treat (NNT) was computed as the reciprocal of the effective rate. Funnel plot was performed to evaluate if publication bias existed. In addition, the overall quality of grading evaluation for the review of evidence was calculated using GRADEprofiler version 3.6 which includes the elements of GRADE criteria such as study design, risk of bias, inconsistency, indirectness, imprecision, and publication bias.

## Results

### Description of studies

**Fig 1:**
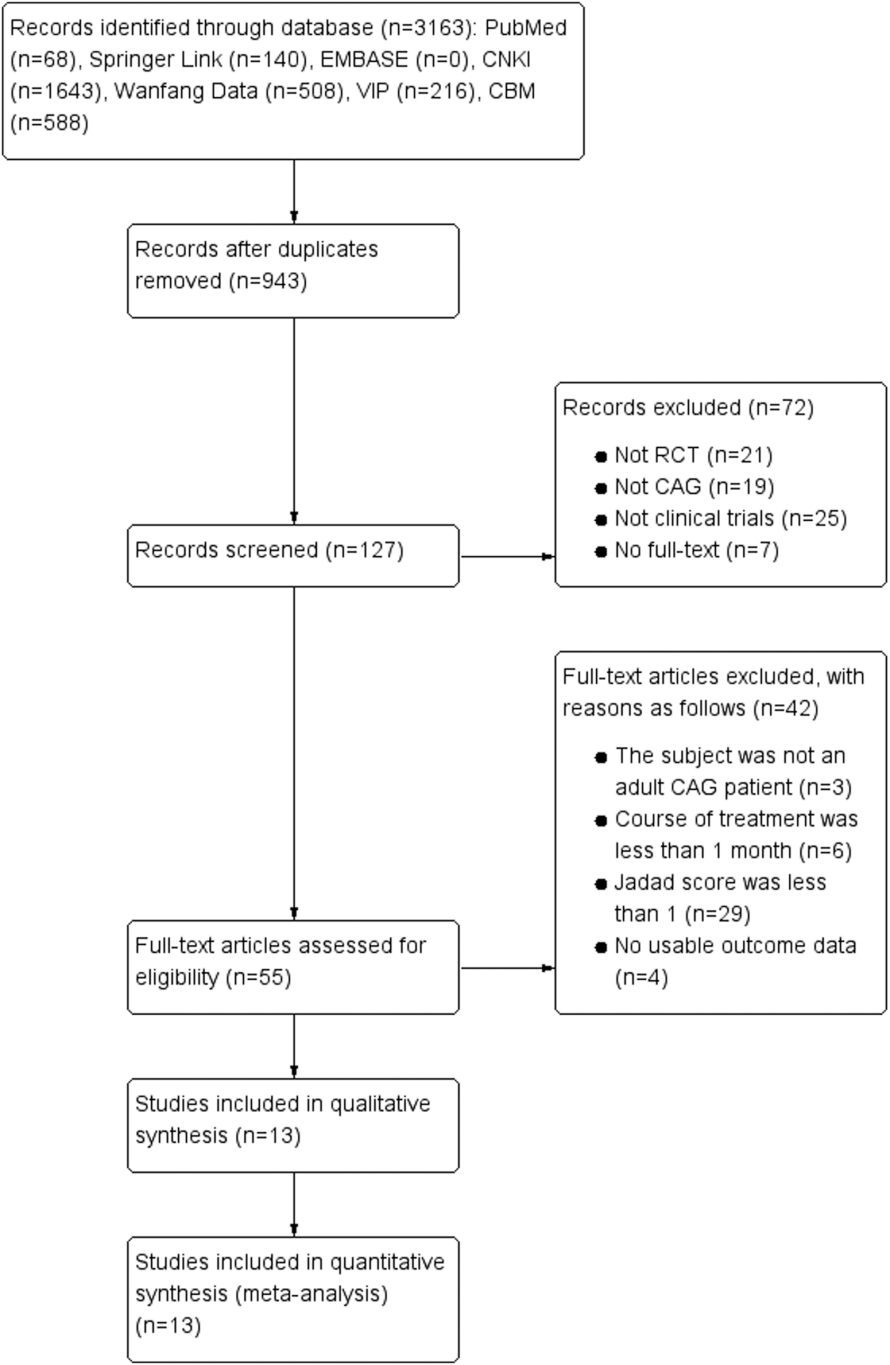
Flow chart of the process for literature retrieval.

**Fig 2:**
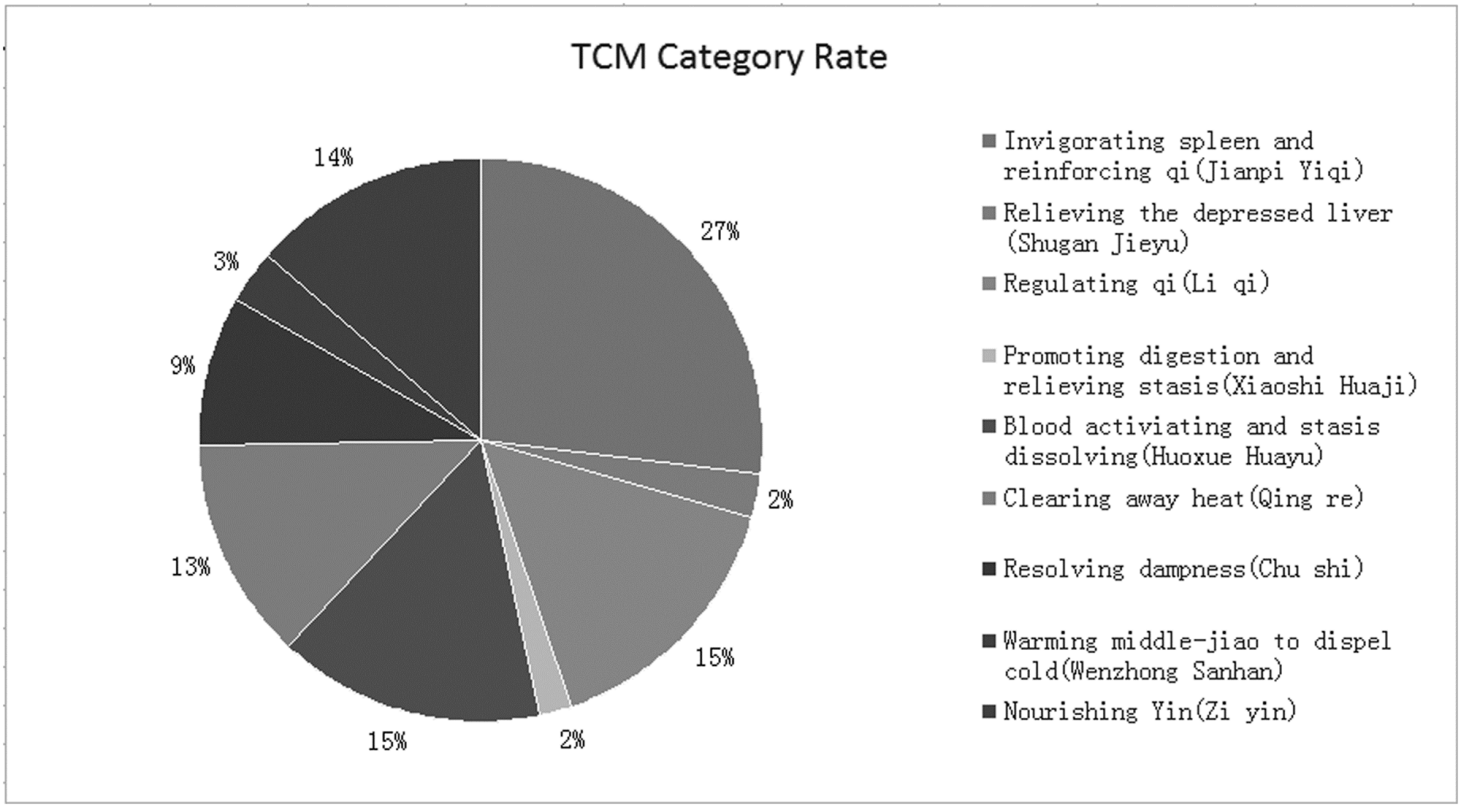
TCM category rate.

A total of 3163 relevant studies were obtained based on the search strategy and screened records. After further reviewing, 13 randomized clinical trials (RCTs) (N = 1119) satisfied all of the criteria and were included in this meta-analysis [8–20]. As for the grey literature, either their data did not meet our criteria or no response had been returned. In addition, although we also searched the EMBASE database which had many latest gray literatures such as meeting abstracts or latest literatures, none of them satisfied our criteria. The flow chart of literature search process was shown in Fig 1 (Flow chart of the process for literature retrieval). In addition, 13 studies were single centre trials and published in Chinese. Sample size was between 53 [16] and 130 [19]. The ages of participants were from 21 to 75 years old. The courses of disease ranged from 1 month to 26 years. The durations were from 4 weeks [13] to 24 weeks [14]. What’s more, the interventions between experiment groups and control groups included the following: JYT versus western medicines (4 trials) [8, 11, 16, 19] and JYT + western medicines versus western medicines (7 trials) [9, 10, 12, 13, 17, 18, 20]. The characteristics of the included studies were described in Table 1. The constituents of herbal formulae were listed in Table 2. Frequencies of usage and distribution in TCM were observed in Table 3. In addition, Chinese herbs classification can be found in Table 4. We can conclude from Tables 3 and 4 that in the all TCM categories, the proportion of invigorating spleen and reinforcing qi (Jianpi Yiqi) was 27.0%, which was the highest frequency among ten kinds of different herbs (Fig 2. TCM category rate).

**Table 1:** Characteristics of the studies included in the meta-analysis.

Study ID (First Author, Year)	Whether by Hp infection	Type of syndrome	Sex		Sample Size (E/C)	Age (years)	Course of disease (years)	Intervention		Duration (weeks)	Outcome measures
			Male (E/C)	Female (E/C)				E	C		
Xu 2016 (8)	Yes	Deficiency cold of spleen and stomach	21/18	22/17	39/39	39–74	1–26	Huangqi Jianzhong decoction, 1 dose/d, b.i.d	Vatacoenayme tablets, 3g, t.i.d	8	A+B+C+D+E+F+G+H
Liang et al. 2016 (9)	No	N.R	21/27	19/29	48/48	**E**:44.3±5.7 **C**:42.6±6.6	**E**:5.36±2.1 **C**:5.12±2.53	Yiqi Huoxue Yangyin Formula, 1 dose/d, b.i.d + Western medicine	Western medicine: Lansoprazole 15mg, Amoxicillin, 1000mg, Clarithromycin, 500mg	12	A+K+M+P+S+T
Ma 2015 (10)	Yes	Deficiency cold of spleen and stomach	20/15	18/17	35/35	23–75	1–15	Jianpi Tongluo Soup, 1 dose/d, 300ml, b.i.d + Western medicine	Western medicine: Omeprazole, 20mg, qd; (Amoxicillin, 2g, Clarithromycin, 1g, b.i.d(10d for a course))	12	A+K+L+M+N+O
Peng et al. 2015 (11)	Yes	N.R	31/24	35/20	55/55	24–71	2/3-14/3	Yiqiyangyin decoction, 1 dose/d, 200ml, b.i.d	H.pylori positive I colloidal bismuth pectin, 300mg, t.i.d, serving two weeks after stopping; Amoxicillin, 3g, t.i.d; Omeprazole, 40mg, b.i.d; Clarithromycin, 1g, b.i.d	8	A
Wang ZX 2015 (12)	No	N.R	20/14	21/23	34/34	25–68	1–11	Prescription for Invigorating Spleen and Stomach, 1dose/d, 400ml, b.i.d + Western medicine	Western medicine: Vatacoenayme tablets, 12 tablets, t.i.d	12	A+B+C+D+E+F
Wang YY 2015 (13)	No	Qi deficiency and blood stasis	13/17	17/13	30/30	**E**:50±6.4 **C**:51±7.2	1–16	Yiqi Huoxue method, 1dose/d, 300ml, b.i.d + Western medicine	Western medicine: Furazolidone 100mg, t.i.d or q.i.d	4	A+B+D+E+G
Zhou et al. 2015 (14)	No	Disharmony between liver and stomach or the stomach-yin of deficiency or stagnated blood of stomach meridian	23/27	24/26	50/50	28–65	3/5-20	Chinese drugs for strengthening Pi, harmonizing Wei, and dispersing blood stasis, 100ml, qd	Folic acid tablets, 30mg, t.i.d	24	P+Q+R+S
Lu et al. 2014 (15)	Yes	N.R	29/31	30/30	60/60	26–70	3–14	Yiqi Yangwei Decotion, 1dose/d, 100ml, b.i.d + Western medicine	Western medicine: Omeprazole 40mg/d, Amoxicillin, 1g, Clarithromycin, 0.5g; b.i.d	12	B+C+D+F+G+P+Q+U
Zhang et al. 2014 (16)	Yes	N.R	14/13	14/12	27/26	32–67	1/6-3/2	Invigorating Blood Circulation to Weak Suppression Soup, 1 dose/d, 400ml, b.i.d	Vatacoenayme tablets, 2.4g, t.i.d; Domperidone, 60mg, t.i.d; Amoxicillin, 15g, t.i.d	12	A
Li 2014 (17)	No	N.R	31/19	33/17	50/50	24–64	1/4-4	Yiwei decoction, b.i.d + Western medicine	Western medicine: Metronidazole 400mg, lansoprazole 30mg, levofloxacin 200mg, b.i.d	8	A
Wang et al. 2013 (18)	Yes	N.R	13/19	14/18	32/32	21–69	21/50-22	Yiqi Huoxue Yangyin method, 1dose/d, 1000ml, b.i.d + Western medicine	Western medicine: Triple therapy (Lansoprazole, 15mg, b.i.d; Amoxicillin, 1000mg, b.i.d; Clarithromycin, 500mg, b.i.d); HP negative antacids, mucosal repair agent symptomatic treatment	8	A
Liu 2013 (19)	No	N.R	36/32	29/33	68/62	30–61	1/12-6	Yiqi Huoxue Huazhuo Jiedu Decoction, 1 dose/d	Vatacoenayme tablets, 2.4g, t.i.d	12	A
Chen et al. 2010 (20)	Yes	Weakness of spleen and stomach	21/15	17/16	36/33	22–65	**E**:6.35±1.96 **C**:6.30±1.98	Traditional Chinese herbal formula, 1 dose/d, 300ml, b.i.d + Western medicine	Western medicine: Colloidal bismuth pectin, 150mg, t.i.d; Berberine tablets, 0.2g, t.i.d; Vatacoenayme tablets, 4 tablets, t.i.d	8	A+E+F+G+I+J

Annotation: A = effective rate; B = stomachache; C = stomach distention; D = belching and acid reflux; E = fatigue; F = poor appetite; G = loose stool; H = cold limbs; I = epigastric distention; J = epigastric pain; K = atrophy; L = atypical hyperplasia; M = intestinal metaplasia; N = chronic inflammation; O = activity; P = endoscopic efficacy; Q = histopathological efficacy; R = the optical density value of gastric mucosal HSP70; S = TCM symptoms and signs efficacy; T = hemorheology indexes; U = Hp eradication rate; Hp = *Helicobacter pylori*; TCM = traditional Chinese medicine; N.R = not reported; **E** = experiment group; **C** = control group.

**Table 2:** The ingredients of each formula.

Author	Ingredients of each formula			
Xu 2016 (8)	*Astragalus membranaceus* (Huang Qi) 15g	*Cynanchum otophyllum* (Bai Shao) 15g	*Polygonatum odoratum* (Yu Zhu) 15g	*Radix Glycyrrhizae preparata* (Zhi Gan Cao) 15g
	*Aconitum carmichaeli Debx* (Fu Zi) 10g	*Amomum villosum Lour* (Sha Ren) 10g	*Hippophae rhamnoides L* (Yi Tang) 10g	*Cinnamomum cassia Presl* (Gui Zhi) 6g
	*Aucklandia lappa Decne* (Mu Xiang) 6g	*Zingiber officinale Rose* (Sheng Jiang) 6g	*Ziziphus jujuba Mill* (Da Zao) 6g	
Liang et al. 2016 (9)	*Astragalus membranaceus* (Huang Qi) 15g	*Codonopsis pilosula (Franch*.*) Nannf* (Dang Shen) 15g	*Aaugellica sinensis(Oliv) Diels* (Dang Gui) 9g	*Citrus reticulata Blanco* (Chen Pi) 9g
	*Ophiopogon japonicus(Thunb*.*) Ker-Gawl* (Mai Dong) 15g	*Atractylodes macrocephala Koidz* (Chao Bai Zhu) 9g	*Polygonatum odoratum* (Yu Zhu) 12g	*Glehnia littoralis Fr*. *Schmidt ex Miq* (Bei Sha Shen) 12g
	*Citrus aurantium L* (Zhi Qiao) 9g	*Citrus medica L*.*Var*. *Sarcodactylis Swingle* (Fo Shou) 9g	*Panax notoginseng (Burk*.*) F*. *H*. *Chen* (San Qi) 6g	*Radix Glycyrrhizae preparata* (Zhi Gan Cao) 3g
	*Paeonia lactiflora Pall*. (Chi Shao)12g	*Curcuma wenyujin Y*.*H*.*Chen et C*.*Ling* (Yu Jin)12g		
Ma 2015 (10)	*Poria cocos (Schw*.*) Wol*f (Fu Lin) 20g	*Astragalus membranaceus* (Huang Qi) 20g	*Atractylodes macrocephala Koidz*. (Bai Zhu) 15g	*Salvia miltiorrhiza Bge* (Dan Shen) 20g
	*Codonopsis pilosula (Franch*.*) Nannf* (Dang Shen) 15g	*Pinellia ternata(Thunb) Breit* (Fa Ban Xia) 9g	*Amomum villosum Lour* (Sha Ren) 6g	*Cinnamomum cassia Presl* (Gui Zhi) 6g
	*Radix Glycyrrhizae preparata* (Zhi Gan Cao) 6g	*Zingiber officinale Rosc* (Gan Jiang) 6g		
Peng et al. 2015 (11)	*Salvia miltiorrhiza Bge* (Dan Shen) 10g	*Cynanchum otophyllum* (Bai Shao) 10g	*Ophiopogon japonicus (Thunb*.*) Ker-Gawl* (Mai Dong) 10g	*Glehnia littoralis Fr*. *Schmidt ex Miq* (Sha Shen) 10g
	*Aaugellica sinensis(Oliv) Diels* (Dang Gui) 10g	*Pinellia ternata(Thunb) Breit* (Jiang Ban Xia) 9g	*Scutellaria barbataD*.*Don* (Ban Zhi Lian) 9g	*Radix Glycyrrhizae preparata* (Gan Cao) 9g
	*Citrus aurantium L* (Zhi Qiao) 9g	*Solanum nigrum L* (Long Kui) 9g	*Dolichos lablab L* (Chao Bian Dou) 15g	*Oldenlandia diffusa (willd*.*) Rox* (Bai Hua She She Cao)15g
	*Codonopsis pilosula (Franch*.*) Nannf* (Dang Shen) 15g			
Wang ZX 2015 (12)	*Codonopsis pilosula (Franch*.*) Nannf* (Dang Shen) 30g	*Astragalus membranaceus* (Huang Qi) 30g	*Poria cocos (Schw*.*) Wol*f (Fu Lin) 15g	*Atractylodes macrocephala Koidz* (Bai Zhu) 12g
	*Dioscorea opposita Thunb* (Shan Yao) 20g	*Salvia miltiorrhiza Bge* (Dan Shen) 15g	*Rehmannia glutinosa Libosch* (Sheng Di Huang) 30g	*Aaugellica sinensis(Oliv) Diels* (Dang Gui) 20g
	*Citrus aurantium L* (Zhi Shi) 10g	*Pinellia ternate (Thunb) Breit* (Ban Xia) 10g	*Citrus reticulata Blanco* (Chen Pi) 10g	*Oldenlandia diffusa (willd*.*) Roxb* (Bai Hua She She Cao) 30g
	*Radix Glycyrrhizae preparata* (Zhi Gan Cao) 6g			
Wang YY 2015 (13)	*Astragalus membranaceus* (Huang Qi) 30g	*Panax quinquefolium L* (Xi Yang Shen)10g	*Prunus persica (L*.*)* Batsch (Tao Ren) 6g	*Carthamus tinctorius L* (Hong Hua) 6g
	*Bupleurum chinensis DC*. (Chai Hu) 12g	*Aucklandia lappa Decne* (Mu Xiang) 6g	*Bletilla striata (Thunb*.*) Reichb*.*F* (Bai Ji) 9g	*Ligusticum chuanxiong Hort* (Chuan Xiong) 6g
	*Panax notoginseng (Burk*.*) F*. *H*.*Chen* (Tian San Qi) 9g	*Gallus gallus domesticus Brisson* (Ji Nei Jin) 15g	*Coptis chinensis Franch* (Huang Lian) 3g	*Citrus reticulata Blanco* (Chen Pi) 15g
Zhou et al. 2015 (14)	*Astragalus membranaceus* (Huang Qi) 12g	*Corydalis yanhusuo W*.*T*.*Wang* (Yan Hu Suo) 10g	*Cyperus rotundus L* (Xiang Fu) 10g	*Crataegus pinnatifida Bge*. *var*. *major N*.*E*.*Br* (Shan Zha) 12g
	*Cynanchum otophyllum* (Bai Shao) 10g	*Glehnia littoralis Fr*. *Schmidt ex Miq* (Bei Sha Shen) 10g	*Ophiopogon japonicus (Thunb*.*) Ker-Gawl* (Mai Dong) 10g	*Salvia miltiorrhiza Bge* (Dan Shen) 12g
	*Radix Glycyrrhizae preparata* (Zhi Gan Cao) 6g			
Lu et al. 2014 (15)	*Astragalus membranaceus* (Sheng Huang Qi) 20g	*Aaugellica sinensis (Oliv) Diels* (Dang Gui) 15g	*Rehmannia glutinosa Libosch* (Sheng Di Huang) 15dig	*Taraxacum mongolicum Hand*. *-Mazz* (Pu Gong Ying) 10g
	*Radix Glycyrrhizae preparata* (Gan Cao) 10g	*Angelica dahurica (Fisch*.*ex Hoffm*.*) Benth*.*et Hook*.*f*. (Bai zhi) 10g	*Lysimachia christinae Hance* (Jin Qian Cao) 8g	*Lycium chinense Mil1*. (Di Gu Pi) 15g
	*A*.*kravanh Pierre ex Gagnep*. (Dou Kou) 12g	*Dendrobium loddigesii Rolfe*. (Shi Hu) 18g	*Nelumbo nucifera Gaertn*. (He Geng) 10g	
Zhang et al. 2014 (16)	*Codonopsis pilosula (Franch*.*) Nannf* (Dang Shen) 20g	*Atractylodes lancea* (*Thunb*.) *DC* (Cang Zhu) 10g	*Atractylodes macrocephala Koidz* (Bai Zhu) 10g	*Poria cocos (Schw*.*) Wol*f (Fu Lin) 15g
	*Radix Glycyrrhizae preparata* (Zhi Gan Cao) 10g	*Curcuma phaeocaulis Val* (E Zhu) 10g	*Salvia miltiorrhiza Bge* (Dan Shen) 15g	*Pinellia ternate (Thunb) Breit* (Jiang Ban Xia) 10g
	*Citrus reticulata Blanco* (Chen Pi) 6g			
Li 2014 (17)	*Ophiopogon japonicus (Thunb*.*) Ker-Gawl* (Mai Dong) 8g	*Glehnia littoralis Fr*. *Schmidt ex Miq* (Sha Shen) 12g	*Aaugellica sinensis (Oliv) Diels* (Dang Gui) 10g	*Scutellaria barbataD*.*Don* (Ban Zhi Lian) 8g
	*Coptis chinensis Franch*. (Huang Lian)6g	*Dolichos lablab L* (Chao Bian Dou) 12g	*Cynanchum otophyllum* (Bai Shao) 12g	*Pinellia ternate (Thunb) Breit* (Jiang Ban Xia) 8g
	*Radix Glycyrrhizae preparata* (Gan Cao) 8g	*Oldenlandia diffusa (willd*.*) Roxb* (Bai Hua She She Cao) 14g		
Wang et al. 2013 (18)	*Cynanchum otophyllum* (Bai Shao) 10g	*Salvia miltiorrhiza Bge* (Dan Shen) 10g	*Glehnia littoralis Fr*. *Schmidt ex Miq* (Sha Shen) 10g	*Ophiopogon japonicus (Thunb*.*) Ker-Gawl* (Mai Dong) 10g
	*Aaugellica sinensis (Oliv) Diels* (Dang Gui) 10g	*Scutellaria barbataD*. *Don* (Ban Zhi Lian) 9g	*Pinellia ternate (Thunb) Breit* (Jiang Ban Xia) 9g	*Solanum nigrum L* (Long Kui) 9g
	*Radix Glycyrrhizae preparata* (Gan Cao) 9g	*Citrus aurantium L* (Zhi Qiao) 9g	*Codonopsis pilosula (Franch*.*) Nannf* (Dang Shen) 15g	*Dolichos lablab L* (Chao Bian Dou) 15g
	*Oldenlandia diffusa (willd*.*) Roxb* (Bai Hua She She Cao) 15g			
Liu 2013 (19)	*Astragalus membranaceus* (Huang Qi) 30g	*Codonopsis pilosula (Franch*.*) Nannf* (Dang Shen) 30g	*Atractylodes macrocephala Koidz* (Chao Bai Zhu) 10g	*Poria cocos (Schw*.*)Wol*f (Fu Lin) 10g,
	*Citrus reticulata Blanco* (Chen Pi) 10g,	*Citrus aurantium L* (Zhi Shi) 10g	*Taraxacum mongolicum Hand*. *-Mazz* (Pu Gong Ying) 30g	*Oldenlandia diffusa (willd*.*) Roxb* (Bai Hua She She Cao) 30g
	*Aaugellica sinensis(Oliv) Diels* (Dang Gui) 10g	*Bletilla striata (Thunb*.*) Reichb*. *F*. (Bai Ji) 30g	*Corydalis yanhusuo W*.*T*.*Wang* (Yan Hu Suo) 10g	*Pinellia ternata(Thunb) Breit*. (Ban Xia) 10g
	*Coix lacryma-jobi L*.*var*.*ma-yuen (Roman*.*) Stapf* (Yi Yi Ren) 30g	*Gallus gallus domesticus Brisson* (Ji Nei Jin) 10g	*Dioscorea opposita Thunb* (Shan Yao) 25g	*Aucklandia lappa Decne* (Mu Xiang) 10g
	*Scutellaria barbataD*.*Don* (Ban Zhi Lian) 10g			
Chen et al. 2010 (20)	*Codonopsis pilosula (Franch*.*) Nannf* (Dang Shen) 20g	*Dioscorea opposita Thunb*. (Shan Yao) 20g	*Astragalus membranaceus* (Zhi Huang Qi) 15g	*Poria cocos (Schw*.*) Wol*f (Fu Lin) 15g
	*Atractylodes macrocephala Koidz*. (Chao Bai Zhu) 10g	*Coptis chinensis Franch*. (Huang Lian)6g	*Aaugellica sinensis(Oliv) Diels* (Dang Gui) 10g	*Citrus medica L*.*Var*. *Sarcodactylis Swingle* (Fo Shou) 10g
	*Amomum villosum Lour* (Sha Ren) 6g	*Glehnia littoralis Fr*. *Schmidt ex Miq* (Sha Shen) 6g	*Evodia rutaecarpa (Juss*.*) Benth*. (Wu Zhu Yu) 5g	*Radix Glycyrrhizae preparata* (Zhi Gan Cao) 5g
	*Panax notoginseng (Burk*.*) F*. *H*. *Chen* (San Qi Fen) 3g			

**Table 3:** Frequencies of usage and distribution in TCM.

Chinese herbs	Frequency	Rate(%)	Chinese herbs	Frequency	Rate(%)
*Radix Glycyrrhizae preparata*(Gan Cao)	11	7.1	*Cinnamomum cassia Presl*(Gui Zhi)	2	1.3
*Astragalus membranaceus*(Huang Qi)	9	5.8	*Corydalis yanhusuo W*.*T*.*Wang* (Yan Hu Suo)	2	1.3
*Codonopsis pilosula (Franch*.*)Nannf*.(Dang Shen)	8	5.2	*Bletilla striata (Thunb*.*) Reichb*. *F*.(Bai Ji)	2	1.3
*Aaugellica sinensis(Oliv) Diels*.(Dang Gui)	8	5.2	*Lycium chinense Mil1*.(Di Gu Pi)	1	0.6
*Oldenlandia diffusa (willd*.*) Roxb*.(Bai Hua She She Cao)	6	3.8	*Evodia rutaecarpa (Juss*.*) Benth*.(Wu Zhu Yu)	1	0.6
*Salvia miltiorrhiza Bge*.(Dan Shen)	6	3.8	*Lysimachia christinae Hance*(Jin Qian Cao)	1	0.6
*Pinellia ternata(Thunb) Breit*.(Ban Xia)	6	3.8	*Coix lacryma-jobi L*.*var*.*ma-yuen (Roman*.*) Stapf*(Yi Yi Ren)	1	0.6
*Glehnia littoralis Fr*. *Schmidt ex Miq*.(Sha Shen)	6	3.8	*Ligusticum chuanxiong Hort*.(Chuan Xiong)	1	0.6
*Atractylodes macrocephala Koidz*.(Bai Zhu)	6	3.8	*Bupleurum chinensis DC*.(Chai Hu)	1	0.6
*Cynanchum otophyllum*(Bai Shao)	5	3.2	*Carthamus tinctorius L*.(Hong Hua)	1	0.6
*Poria cocos (Schw*.*)Wol*f(Fu Lin)	5	3.2	*Prunus persica(L*.*)*Batsch(Tao Ren)	1	0.6
*Ophiopogon japonicus(Thunb*.*)Ker-Gawl*.(Mai Dong)	5	3.2	*Panax quinquefolium L*.(Xi Yang Shen)	1	0.6
*Citrus reticulata Blanco*(Chen Pi)	5	3.2	*Dendrobium loddigesii Rolfe*.(Shi Hu)	1	0.6
*Scutellaria barbataD*.*Don*.(Ban Zhi Lian)	4	2.6	*Zingiber officinale Rosc*.(Gan Jiang)	1	0.6
*Dolichos lablab L*.(Bian Dou)	3	1.9	*Curcuma phaeocaulis Val*.(E Zhu)	1	0.6
*Coptis chinensis Franch*.(Huang Lian)	3	1.9	*Curcuma wenyujin Y*.*H*.*Chen et C*.*Ling*(Yu Jin)	1	0.6
*Dioscorea opposita Thunb*.(Shan Yao)	3	1.9	*Atractylodes lancea* (*Thunb*.) *DC*.(Cang Zhu)	1	0.6
*Amomum villosum Lour*(Sha Ren)	3	1.9	*Aconitum carmichaeli Debx*(Fu Zi)	1	0.6
*Aucklandia lappa Decne*(Mu Xiang)	3	1.9	*Hippophae rhamnoides L*(Yi Tang)	1	0.6
*Citrus aurantium L*.(Zhi Qiao)	3	1.9	*Zingiber officinale Rose*(Sheng Jiang)	1	0.6
*Panax notoginseng (Burk*.*) F*. *H*. *Chen*(San Qi)	3	1.9	*Ziziphus jujuba Mill*(Da Zao)	1	0.6
*Gallus gallus domesticus Brisson*(Ji Nei Jin)	2	1.3	*Cyperus rotundus L*.(Xiang Fu)	1	0.6
*Solanum nigrum L*. (Long Kui)	2	1.3	*Crataegus pinnatifida Bge*.*var*.*major N*.*E*.*Br*.(Shan Zha)	1	0.6
*Citrus aurantium L*.(Zhi Shi)	2	1.3	*Paeonia lactiflora Pall*.(Chi Shao)	1	0.6
*Polygonatum odoratum* (Yu Zhu)	2	1.3	*A*.*kravanh Pierre ex Gagnep*.(Dou Kou)	1	0.6
*Rehmannia glutinosa Libosch*.(Sheng Di Huang)	2	1.3	*Angelica dahurica (Fisch*.*ex Hoffm*.*)Benth*.*et Hook*.*f*.(Bai zhi)	1	0.6
*Citrus medica L*. *Var*. *Sarcodactylis Swingle*(Fo Shou)	2	1.3	*Nelumbo nucifera Gaertn*.(He Geng)	1	0.6
*Taraxacum mongolicum Hand*.*-Mazz*.(Pu Gong Ying)	2	1.3			

**Table 4:** Chinese herbs classification.

TCM Category	Chinese herbs			
Invigorating spleenand reinforcing qi(Jianpi Yiqi)	*Radix Glycyrrhizae preparata* (Gan Cao)	*Codonopsis pilosula (Franch*.*)Nannf*. (Dang Shen)	*Astragalus membranaceus* (Huang Qi)	*Ziziphus jujuba Mill* (Da Zao)
	*Atractylodes macrocephala Koidz* (Bai Zhu)	*Dolichos lablab L* (Bian Dou)	*Dioscorea opposita Thunb* (Shan Yao)	*Panax quinquefolium L* (Xi Yang Shen)
	*Atractylodes lancea* (*Thunb*) *DC* (Cang Zhu)	*Hippophae rhamnoides L* (Yi Tang)		
Regulating qi (Li qi)	*Citrus reticulata Blanco* (Chen Pi)	*Citrus aurantium L* (Zhi Qiao)	*Aucklandia lappa Decne* (Mu Xiang)	*Citrus aurantium L* (Zhi Shi)
	*Cyperus rotundus L* (Xiang Fu)	*Amomum villosum Lour* (Sha Ren)	*Citrus medica L*.*Var*. *Sarcodactylis Swingle* (Fo Shou)	*Corydalis yanhusuo W*.*T*.*Wang* (Yan Hu Suo)
	*Cinnamomum cassia Presl* (Gui Zhi)	*Curcuma wenyujin Y*.*H*.*Chen et C*.*Ling* (Yu Jin)	*Nelumbo nucifera Gaertn*. (He Geng)	
Relieving the depressed liver (Shugan Jieyu)	*Bupleurum chinensis DC* (Chai Hu)	*Cyperus rotundus L* (Xiang Fu)	*Citrus medica L*.*Var*. *Sarcodactylis Swingle* (Fo Shou)	
Promoting digestion and relieving stasis (Xiaoshi Huaji)	*Crataegus pinnatifida Bge*.*var*.*major N*.*E*.*Br*. (Shan Zha)	*Gallus gallus domesticus Brisson* (Ji Nei Jin)		
Blood activiatingand stasis dissolving(Huoxue Huayu)	*Ligusticum chuanxiong Hort* (Chuan Xiong)	*Salvia miltiorrhiza Bge* (Dan Shen)	*Curcuma phaeocaulis Val* (E Zhu)	*Aaugellica sinensis(Oliv) Diels* (Dang Gui)
	*Panax notoginseng (Burk*.*) F*. *H*. *Chen* (San Qi)	*Bletilla striata (Thunb*.*) Reichb*.*F* (Bai Ji)	*Carthamus tinctorius L* (Hong Hua)	*Prunus persica(L*.*)* Batsch (Tao Ren)
	*Curcuma wenyujin Y*.*H*.*Chen et C*.*Ling* (Yu Jin)	*Paeonia lactiflora Pall*. (Chi Shao)		
Resolving dampness (Chu shi)	*Pinellia ternata(Thunb) Breit* (Ban Xia)	*Poria cocos (Schw*.*)Wol*f (Fu Lin)	*Atractylodes lancea* (*Thunb*.) *DC* (Cang Zhu)	*Coix lacryma-jobi L*.*var*.*ma-yuen (Roman*.*) Stapf* (Yi Yi Ren)
	*A*.*kravanh Pierre ex Gagnep*. (Dou Kou)			
Clearing away heat (Qing re)	*Coptis chinensis Franch* (Huang Lian)	*Taraxacum mongolicum Hand*.*-Mazz* (Pu Gong Ying)	*Oldenlandia diffusa (willd*.*) Roxb*. (Bai Hua She She Cao)	*Scutellaria barbataD*.*Don* (Ban Zhi Lian)
	*Rehmannia glutinosa Libosch* (Sheng Di Huang)	*Solanum nigrum L*. (Long Kui)	*Lycium chinense Mil1*. (Di Gu Pi)	*Lysimachia christinae Hance* (Jin Qian Cao)
Warming middle-jiao to dispel cold (Wenzhong Sanhan)	*Evodia rutaecarpa (Juss*.*) Benth*. (Wu Zhu Yu)	*Zingiber officinale Rose* (Sheng Jiang)	*Zingiber officinale Rosc*. (Gan Jiang)	*Aconitum carmichaeli Debx* (Fu Zi)
	*Angelica dahurica (Fisch*.*ex Hoffm*.*) Benth*.*et Hook*.*f*. (Bai zhi)			
Nourishing Yin (Zi yin)	*Cynanchum otophyllum* (Bai Shao)	*Glehnia littoralis Fr*. *Schmidt ex Miq*. (Sha Shen)	*Ophiopogon japonicus (Thunb*.*)Ker-Gawl*. (Mai Dong)	*Panax quinquefolium L* (Xi Yang Shen)
	*Rehmannia glutinosa Libosch*. (Sheng Di Huang)	*Polygonatum odoratum* (Yu Zhu)	*Dendrobium loddigesii Rolfe*. (Shi Hu)	

### Risk of bias assessment

**Fig 3:**
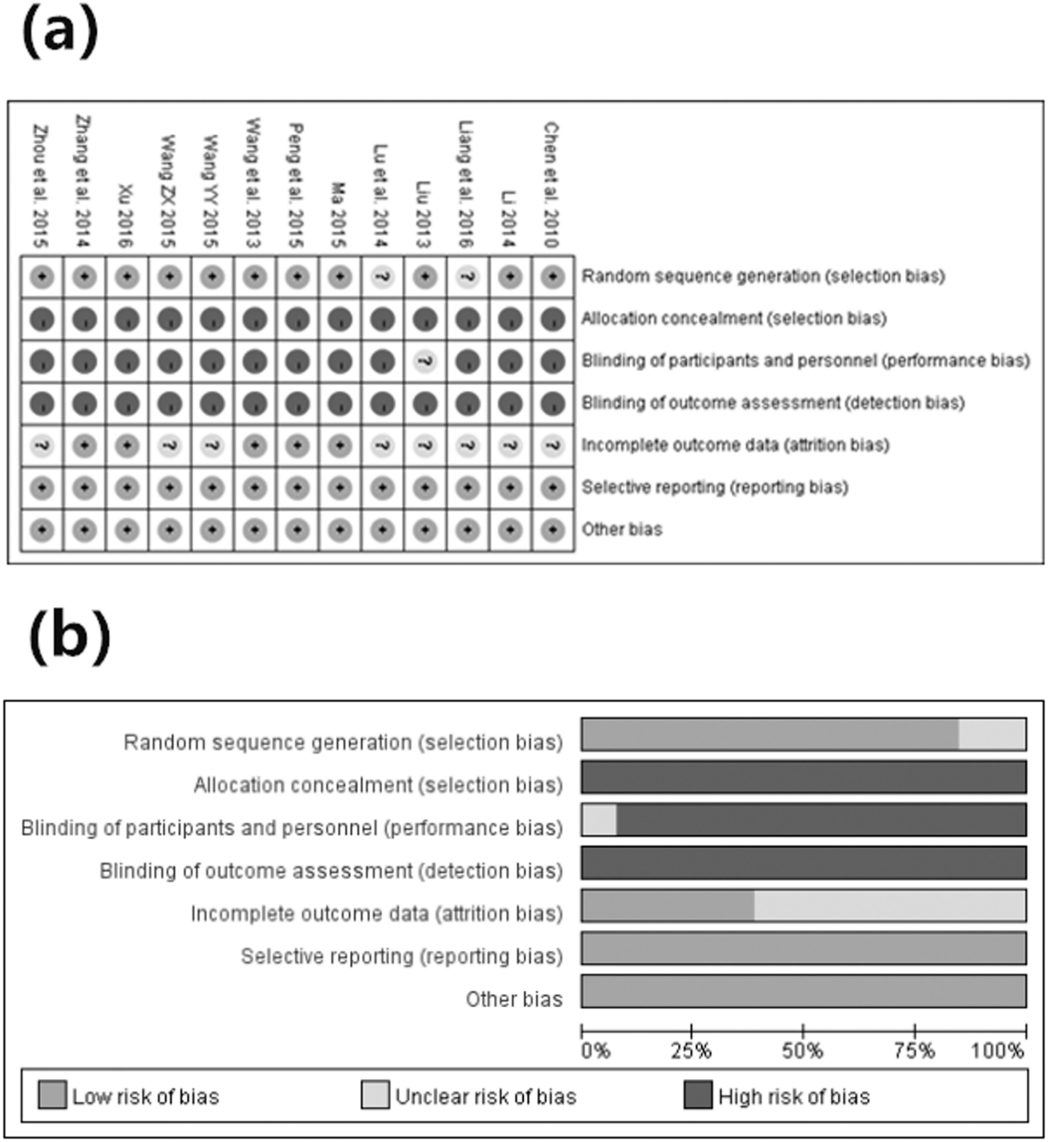
(a) Risk of bias summary. (b) Risk of bias graph.

A description of the assessment of methodological quality of the included trials can be observed in Table 5. Nine studies used a random number table [8, 12–14, 16–20]. Two studies used a method of flipping a coin [10, 11], while the other studies used the word “randomization”, without any explanation of the random-allocation process [9, 15]. Moreover, only one trial reported “single-blind” for the patients [19]. The remaining 12 studies did not mention blinding. Furthermore, none of included trials reported the concealment of allocation. In addition, taking the integrity of outcome data into account, only five trials provided the number of dropouts [8, 10, 11, 16, 18]. However, the missing data were not conducted by intention-to-treat analysis. Because of the relative lack of specific information, it cannot be determined whether implementations were conducted adequately in the process of random sequence generation, blinding or allocation concealment, thus accounting for the high risk in the validity of this review (Fig 3. (a) Risk of bias summary. (b) Risk of bias graph).

**Table 5:** Evaluation of methodological quality of the included studies.

Study ID	Baseline	Randomization	Double Blinding	Withdrawal or dropout	Allocation concealment	Follow-up	Side effects	Jadad scores
Xu 2016 (8)	Comparability	Random number table	N.R	no	N.R	6 months, recurrence(E: 1 case C: 6 cases)	no	3
Liang et al. 2016 (9)	Comparability	Mentioned not described	N.R	N.R	N.R	N.R	N.R	1
Ma 2015 (10)	Comparability	Flipping a coin	N.R	no	N.R	N.R	no	3
Peng et al. 2015 (11)	Comparability	Flipping a coin	N.R	no	N.R	N.R	no	3
Wang ZX 2015 (12)	Comparability	Random number table	N.R	N.R	N.R	N.R	no	2
Wang YY 2015 (13)	Comparability	Random number table	N.R	N.R	N.R	N.R	N.R	2
Zhou et al. 2015 (14)	Comparability	Random number table	N.R	N.R	N.R	N.R	N.R	2
Lu et al. 2014 (15)	Comparability	Mentioned not described	N.R	N.R	N.R	N.R	N.R	1
Zhang et al. 2014 (16)	Comparability	Random number table	N.R	no	N.R	N.R	no	3
Li 2014 (17)	Comparability	Random number table	N.R	N.R	N.R	N.R	N.R	2
Wang et al. 2013 (18)	Comparability	Random number table	N.R	no	N.R	N.R	no	3
Liu 2013 (19)	Comparability	Random number table	Single-blind	N.R	N.R	N.R	N.R	2
Chen et al. 2010 (20)	Comparability	Random number table	N.R	N.R	N.R	N.R	N.R	2

Annotation: N.R = not reported.

### Effects of the interventions: Primary outcomes

#### Comparison of effective rate

Among the included studies, 11 reported the effective rate based on the standards of the Guiding Principles for the Clinical Research of New TCM [21]: Cure, the clinical symptom disappeared; Markedly effective, the clinical symptom markedly improved; Effective, the clinical symptom improved; Ineffective, the clinical symptom did not improve even deteriorate. The effective rate was equal to (the numbers of patients whose clinical symptom improved after intervention divide total numbers of patients) × 100%. For example, the experiment group had 32 patients whose clinical symptom improved after intervention while the control group had 23 patients whose clinical symptom improved after intervention in the Chen et al. study. Moreover, the total numbers of patients in the experiment group were 36 while those in the control group were 33. Therefore, the effective rate in the experiment group was equal to (32 ÷ 36) × 100% while those in the control group was equal to (23 ÷ 33) × 100%. In addition, we did not perform a sensitivity analysis for good homogeneity in primary outcomes.

#### JYT versus western medicine

**Fig 4:**
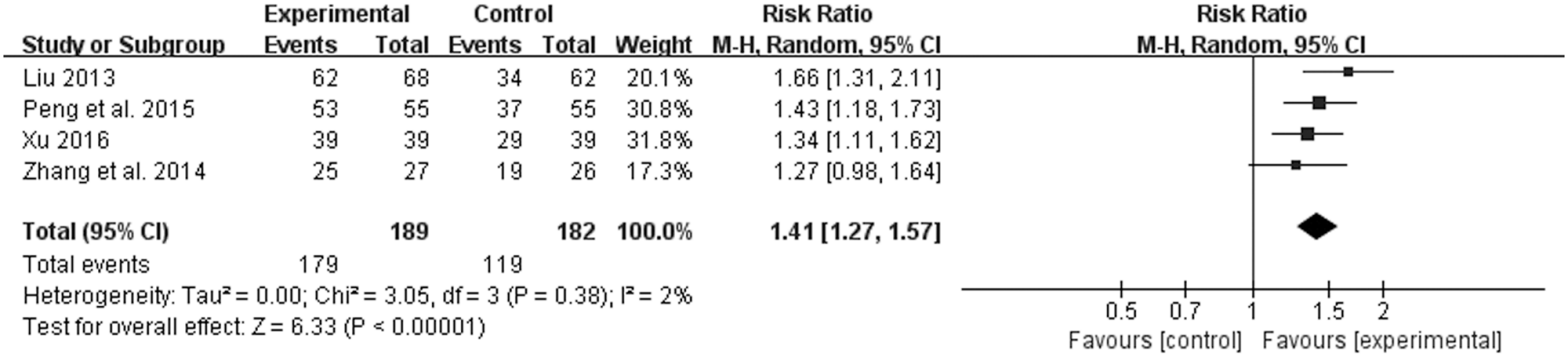
Forest plot of effective rate (random effect model).

**Fig 5:**
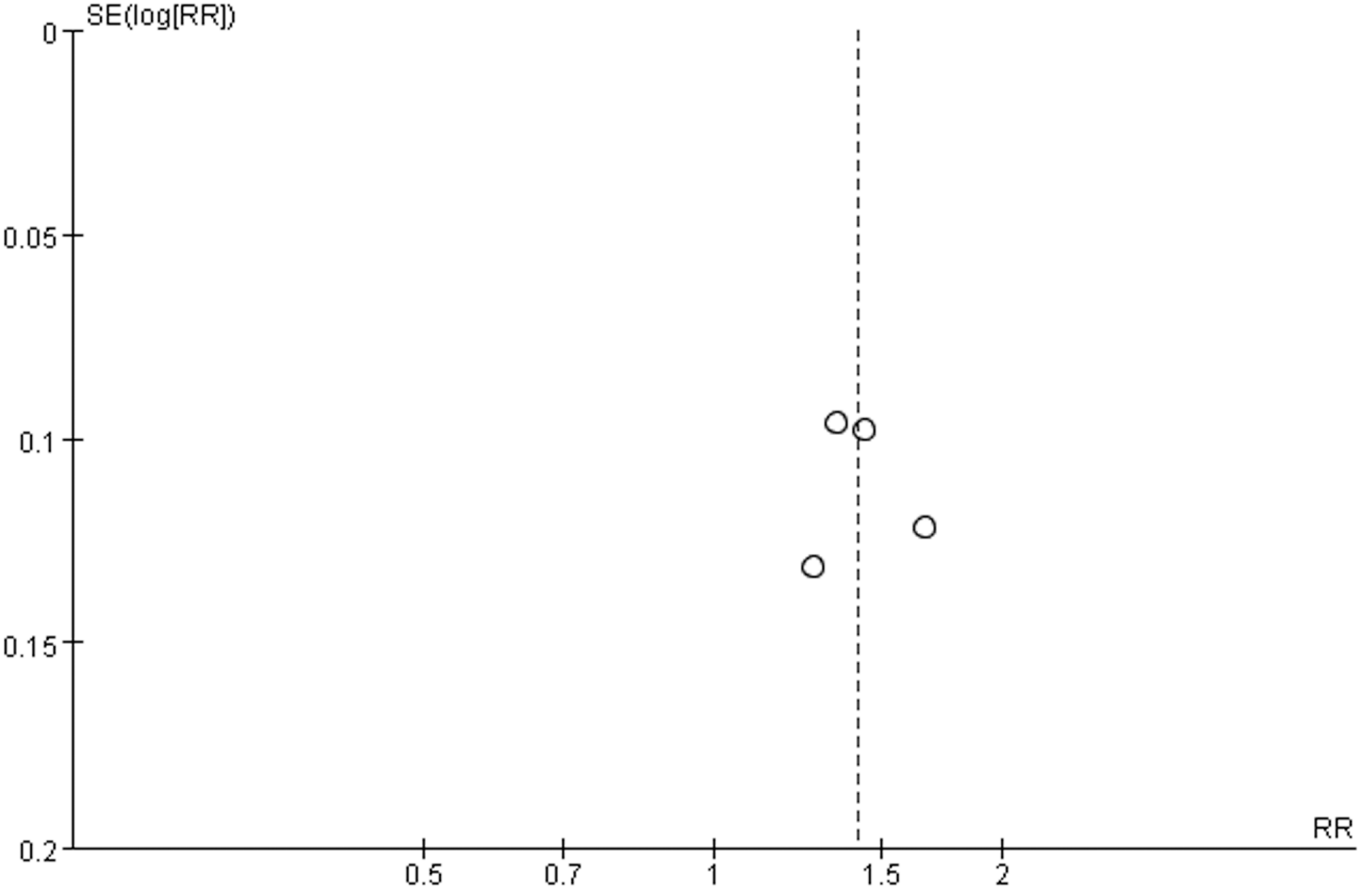
Funnel plot of effective rate.

Four of the thirteen trials including 371 patients (189 in the experiment groups vs. 182 in the control groups) with CAG reported the effective rate [8, 11, 16, 19]. Although the forms of Jianpi Yiqi Theory were decoctions, the doses and methods of preparation and administration were different. Moreover, discrepancies in interventions among control groups were existed. Therefore, a random effect model was applied to estimate pooled effect size despite good homogeneity (*χ*^*2*^ = 3.05, *P* = 0.38, *I*^*2*^ = 2%) (Fig 4. Forest plot of effective rate (random effect model)). JYT showed statistically significant differences in the effective rate compared to western medicine (RR 1.41; 95% CI 1.27, 1.57; *P* < 0.00001) (Fig 4. Forest plot of effective rate (random effect model)). Potential publication bias was identified by asymmetrical funnel plot in Fig 5 (Funnel plot of effective rate).

#### JYT + western medicine versus western medicine

**Fig 6:**
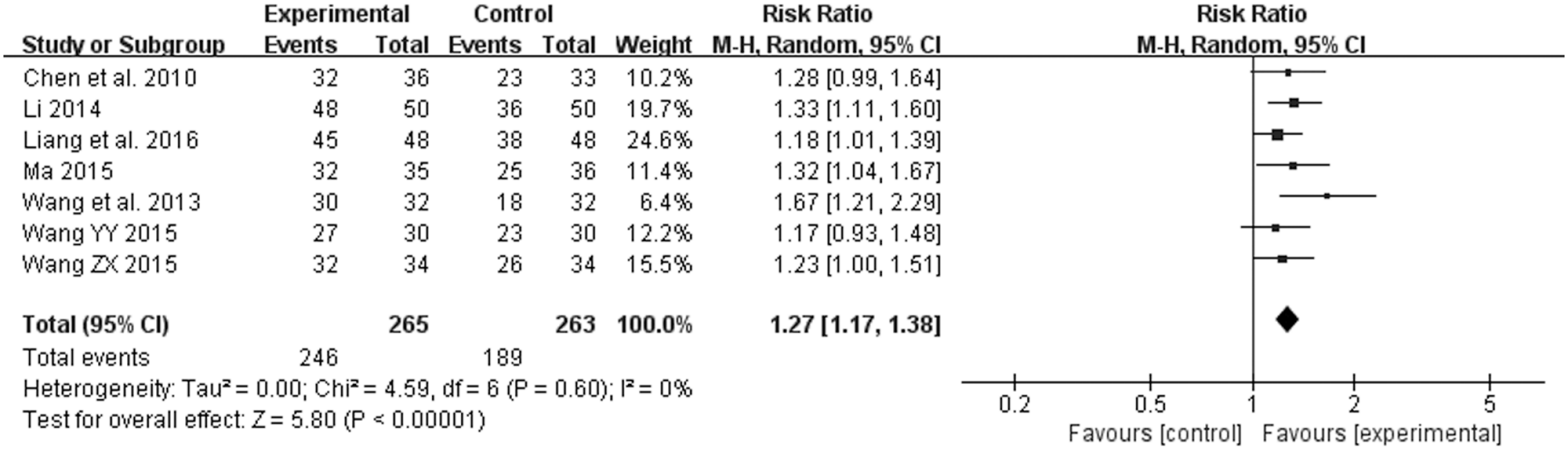
Forest plot of effective rate (random effect model).

**Fig 7:**
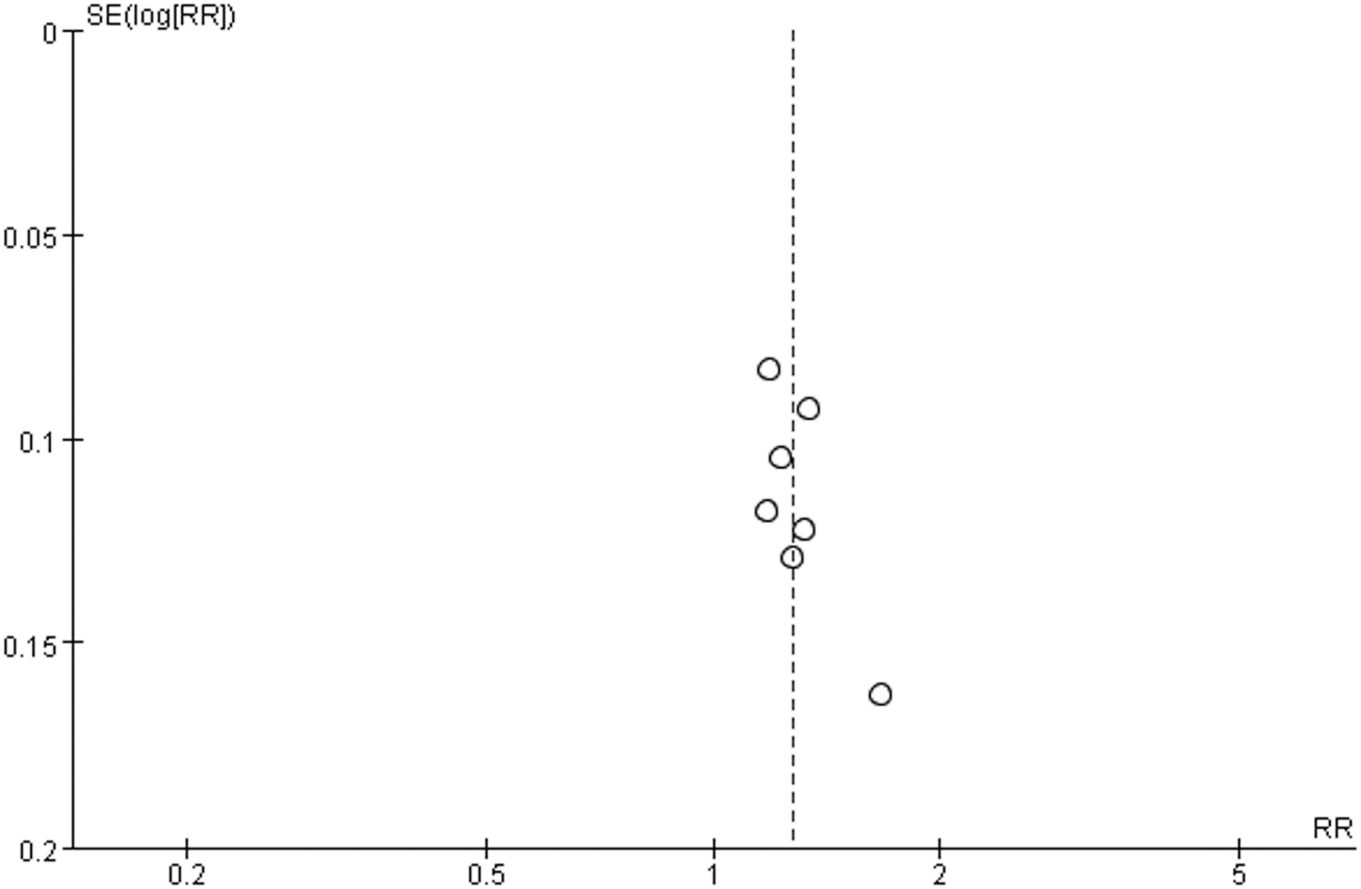
Funnel plot of effective rate.

Seven studies also evaluated the effective rate [9, 10, 12, 13, 17, 18, 20]: of 528 participants, 265 were assigned to the groups of JYT + western medicine, whereas 263 were assigned to the groups of western medicine. Because of the existence of discrepancies in interventions, pooled estimates were conducted by using a model of random effect in spite of no significant heterogeneity (*χ*^*2*^ = 4.59, *P* = 0.60, *I*^*2*^ = 0%) (Fig 6. Forest plot of effective rate (random effect model)). The effective rate of the experiment groups had potentially superior to that of the control groups (RR 1.27; 95% CI 1.17, 1.38; *P* < 0.00001) (Fig 6. Forest plot of effective rate (random effect model)). In addition, one trial reported NNT = 5 (95% CI 2.6, 5000.0) [20]. No evidence of symmetry was observed from the funnel plot in Fig 7 (Funnel plot of effective rate).

#### Subgroup analysis

**Fig 8:**
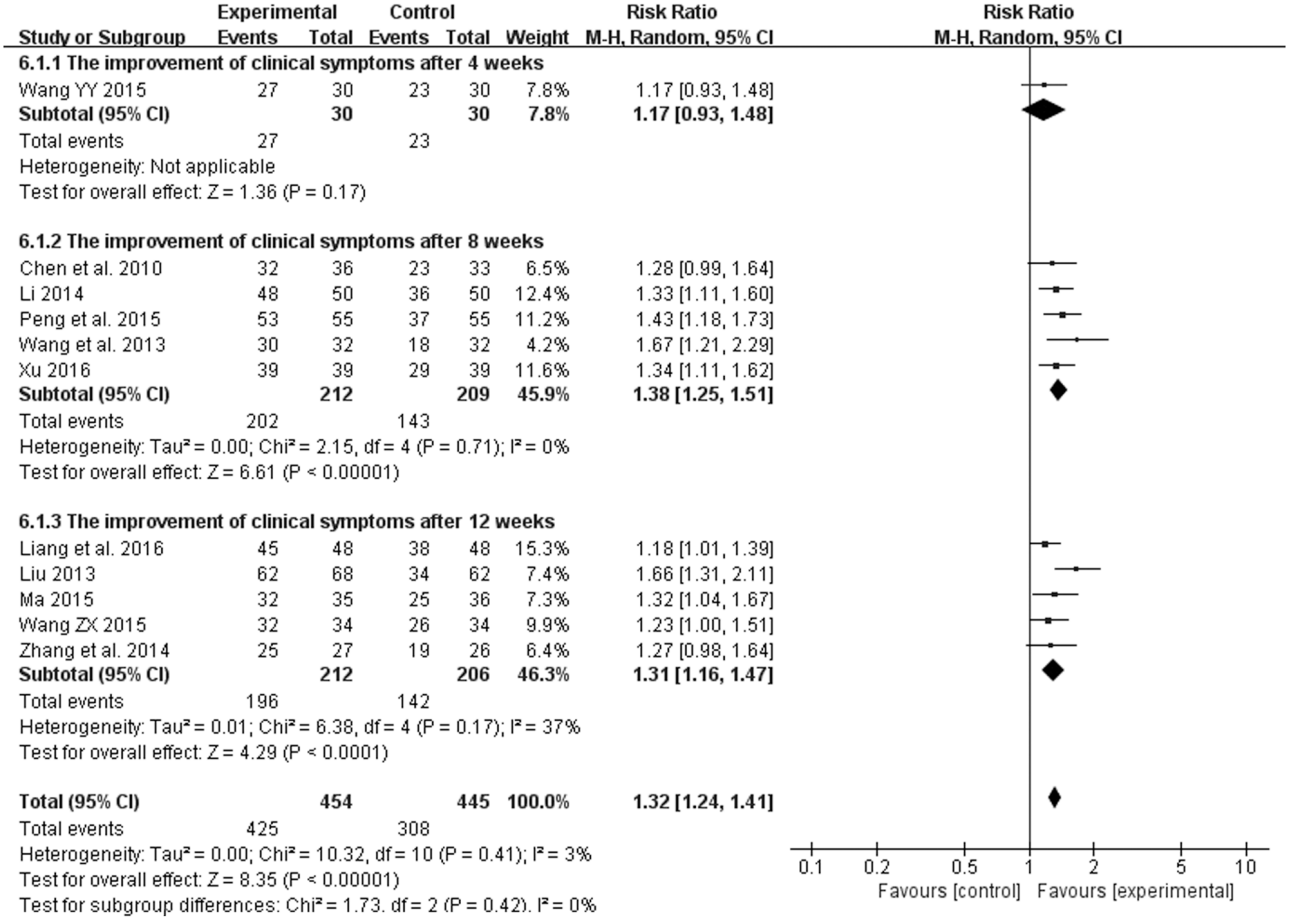
Forest plot of subgroup analysis.

**Fig 9:**
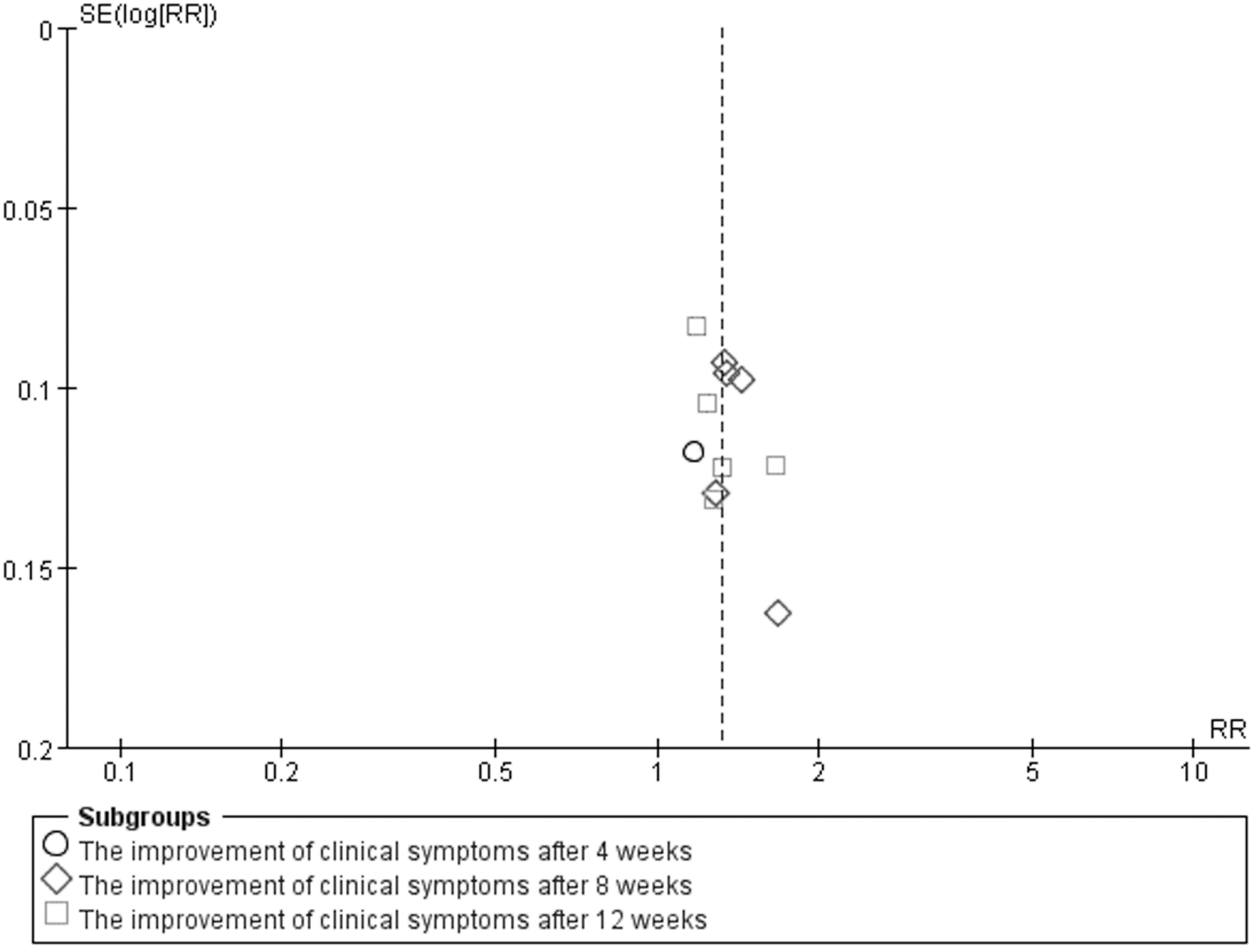
Funnel plot of subgroup analysis.

In addition, because of variability in evaluating point of the effective rate, we conducted subgroup analysis among the included studies using different treatment courses of 4, 8, 12 weeks. Compared with the control groups, the experiment groups were positive effects on the improvement of clinical symptoms after 4 weeks (RR 1.17; 95% CI 0.93, 1.48; *P* = 0.17) in one study [13], 8 weeks (RR 1.38; 95% CI 1.25, 1.51; *P* = 0.71) in five studies [8, 11, 17, 18, 20], 12 weeks (RR 1.31; 95% CI 1.16, 1.47; *P* = 0.17) in five studies [9, 10, 12, 16, 19], and an overall effect (RR 1.32; 95% CI 1.24, 1.41; *P* = 0.41) in Fig 8 (Forest plot of subgroup analysis). A funnel plot analysis of the 11 trials [8–13, 16–20] suggested possible publication bias and inclusion of low quality studies because of a significant asymmetry as shown in Fig 9 (Funnel plot of subgroup analysis).

### Secondary outcomes

#### Improvement of TCM symptoms scores

Of all the included studies, four reported the improvement of stomachache [8, 12, 13, 15], three reported the improvement of stomach distention and belching [8, 12, 15], and three reported the improvement of fatigue [8, 12, 20]. Moreover, all of them were analyzed by a consensus [22] or semiquantitative scoring system. Although discrepancies in scoring system were existed, every study showed that JYT or combined with conventional western medicines can significantly improve these TCM symptoms caused by CAG.

### The treating improvements in endoscopic and histopathologic test results

In the included trials, three reported the treating improvement in endoscopy [9, 14, 15] and two reported that in histopathology [14, 15]. Because of few trials reporting the treating improvements in endoscopic and histopathologic test results, the two items were only qualitatively analyzed. But in the treating improvements in endoscopic and histopathologic test results, the treatment groups had potentially superior to the control groups.

### Hp eradication rate

Although seven of thirteen studies described the situation of Hp infection [8, 10, 11, 15, 16, 18, 20], only one reported Hp eradication rate after treatment [15]. However, the study showed the experiment group had better efficacy than the control group in Hp eradication rate [15].

### Adverse events

Of all the included studies, six reported no adverse reactions during JYT treatment [8, 10–12, 16, 18]. Moreover, the adverse effects of the experiment groups were no different from those of the control groups.

### GRADE evidence of quality

**Fig 10:**
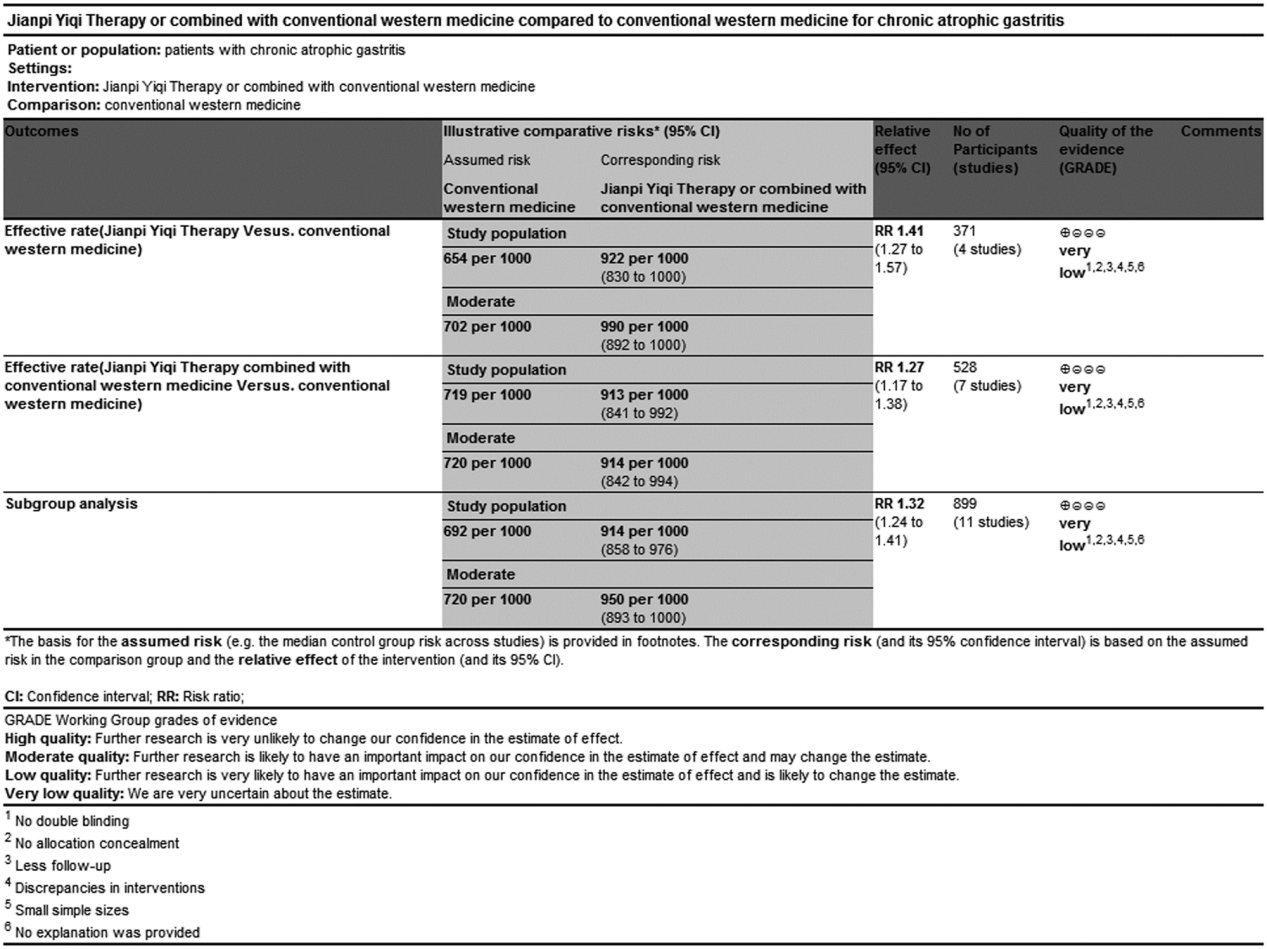
GRADE quality grading evaluation.

GRADEprofiler software, adopted by WHO and the Cochrane collaboration, was used for rating quality of evidence and grading strength of recommendations for this systematic review. GRADE indicated that evidence quality was “Very low”, which may be associated with high risk of bias within RCTs and the relatively small sample sizes of the included studies (Fig 10. GRADE quality grading evaluation) (Table 6).

**Table 6:** GRADE quality grading evaluation.

Quality assessment							No of patients		Effect		Quality	Importance
No of studies	Design	Risk of bias	Inconsistency	Indirectness	Imprecision	Other considerations	Jianpi Yiqi Therapy or combined with conventional western medicine	Conventional western medicine	Relative (95% CI)	Absolute		
Effective rate (Jianpi Yiqi Therapy Vesus. conventional western medicine)												
4	randomised trials	very serious^1,2^	serious^3^	serious^4^	very serious^5^	none^6^	179/189 (94.7%)	119/182 (65.4%)	RR 1.41 (1.27 to 1.57)	268 more per 1000 (from 177 more to 373 more)	ÅOOOVERY LOW	CRITICAL
								70.2%		288 more per 1000 (from 190 more to 400 more)		
Effective rate (Jianpi Yiqi Therapy combined with conventional western medicine Versus. conventional western medicine)												
7	randomised trials	very serious^1,2^	serious^3^	serious^4^	very serious^5^	none^6^	246/265 (92.8%)	189/263 (71.9%)	RR 1.27 (1.17 to 1.38)	194 more per 1000 (from 122 more to 273 more)	ÅOOOVERY LOW	CRITICAL
								72%		194 more per 1000 (from 122 more to 274 more)		
Subgroup analysis (different treatment courses of 4, 8, 12 weeks among the included studies)												
11	randomised trials	very serious^1,2^	serious^3^	serious^4^	very serious^5^	none^6^	425/454 (93.6%)	308/445 (69.2%)	RR 1.32 (1.24 to 1.41)	221 more per 1000 (from 166 more to 284 more)	ÅOOOVERY LOW	CRITICAL
								72%		230 more per 1000 (from 173 more to 295 more)		

## Discussion

This meta-analysis revealed that JYT or JYT + western medicine showed better effective rate than only western medicine and can significantly improve TCM symptoms caused by CAG such as stomachache, stomach distention, belching, fatigue, et al. However, weaknesses were identified in most trials using the Cochrane Collaboration’s risk of bias tool, while the quality level of GRADE evidence classification indicated “Very low”. No serious adverse events were found in the included studies. In addition, because of the Chinese herbal medicines of invigorating spleen and reinforcing qi (Jianpi Yiqi) at high proportion in the treatment of CAG (Fig 2. TCM category rate), it can suggest that JYT was possibly a promising therapy in treating CAG and provided practitioners with important reference value on clinical syndrome differentiations.

The pathogenesis of CAG remains still controversial. Numerous mechanisms indicate that the development of CAG is associated with Hp infection, inflammation, gene, and autoimmune diseases [23–27]. Evidence for the efficacy of JYT for CAG was identified in modern pharmacological studies. Experimental data have showed that Xiangsha Liujunzi Decoction is an effective prescription for harmonizing the spleen and stomach, whose mechanisms are possibly associated with protecting GM, promoting gastric emptying, and inhibiting small intestine peristalsis too fast [28]. Another experiment has proved that Shenxiang Yangwei Powder can evidently promote the blood flow in the GM of rabbits, abate the injury of the GM of the white rats by alcohol, and also have preventive effect on lesions and secretive function of experimental CAG model [29]. In addition, clinical trials have demonstrated that Jianpi Huayu Jiedu therapy can relieve the degree of IM and glandular atrophy of GM, possibly by influencing the expression of the Cyclin E protein in the patients with precancerous lesion of GC, thus preventing the development of premalignant lesion of GC [30]. Weishu Capsule can significantly improve the clinical and pathological changes in the precancerous lesions of CAG via inducing and promoting effect of Weishu Capsule on the differentiation and maturity of IM cells and dysplasia cells, as well as inhibiting and correcting the abnormal proliferation of cells [31]. In a word, JYT may be a multitargeting management for the treatment of CAG, deserving to be studied further in vitro and in vivo.

Several potential limitations of this meta-analysis must be acknowledged. First, the methodological quality of included trials was generally poor. Because of no description of allocation concealment and double blind double dummy, this resulted in potentially high risk of selection bias and detection bias or performance bias. Furthermore, potential publication bias possibly existed because studies with favorable results were more likely to be published. Second, only one study mentioned follow-up and its period was half a year [8]. And considering atrophic gastritis as a chronic recurrent disease, its treatment sessions and follow-up periods should be long enough to evaluate long-term clinical effect of JYT. However, the courses of treatment in the included trials were all less than six months. The durations were too short to assess medium- or long-term efficacy and safety of JYT for CAG. Third, although the forms of JYT were decoctions, the doses and methods of preparation and administration were different. Moreover, discrepancies in interventions among control groups were not separately analyzed. These limitations may result in heterogeneous. Fourth, all of the included studies were based in China, not involving foreign countries. This geographically limited distribution could also result in sampling bias in CAG diagnosis. It was hard to validate that the efficacy of JYT for CAG screening applies to different populations worldwide. Fifth, most of the trials used the effective rate as the primary outcome. This will result in inability to quantitatively evaluate the efficacy of JYT for CAG. As for secondary outcomes, we qualitatively described them because of few studies reported. Therefore, the authenticity of the results awaited further proof. Sixth, although several literatures had reported that Hp eradication could possibly reduce GC risk [32–35], only one included study mentioned Hp eradication rate after treatment. This few recorded difference may potentially bring about unreliable and unbelievable results. Seventh, all of the included trials had single centers and small sample sizes, causing unstable results and inability to truly reflect general trends. Therefore, more rigorous designed RCTs are warranted to evaluate the efficacy of JYT for CAG. Furthermore, it remains urgent to make reporting quality of future research improvement strictly based on Strengthening the Reporting of Observational Studies in Epidemiology (STROBE) or Consolidated Standards of Reporting Trials (CONSORT) statement.

## Conclusions

Evidence from this meta-analysis suggests that JYT as an alternative therapy might be more efficacious than control groups, as well as improve TCM symptoms caused by CAG such as stomachache, stomach distention, belching, fatigue, et al. However, due to small sample size and poor methodological quality in the included trials, further standardized research with multicenter, large-scale, and rigorous design should be required.

## Supporting information

S1 PRISMA Checklist (DOC)

S1 Table The ingredients of each formula. (DOC)

S2 Table Frequencies of usage and distribution in TCM. (DOC)

S3 Table Chinese herbs classification. (DOC)

S4 Table GRADE quality grading evaluation. (DOC)

S1 File A sample search strategy. (DOC)

## References

[1] Kuipers EJ, Klinkenberg-Knol EC, Vandenbroucke-Grauls CM, Appelmelk BJ, Schenk BE, Meuwissen SG. Role of Helicobacter pylori in the pathogenesis of atrophic gastritis. Scand J Gastroenterol Suppl. 1997; 223:28–34. 9200303

[2] de Vries AC, van Grieken NC, Looman CW, Casparie MK, de Vries E, Meijer GA, et al Gastric cancer risk in patients with premalignant gastric lesions: a Nationwide Cohort Study in the Netherlands. Gastroenterology. 2008; 134(4):945–952. doi: 10.1053/j.gastro.2008.01.071 18395075 10.1053/j.gastro.2008.01.071

[3] Park YH and Kim N. Review of atrophic gastritis and intestinal metaplasia as a premalignant lesion of gastric cancer. Journal of Cancer Prevention. 2015; 20(1):25–40. doi: 10.15430/JCP.2015.20.1.25 20(1):25–40 25853101 10.15430/JCP.2015.20.1.25 PMC4384712

[4] Simrén M, Svedlund J, Posserud I, Björnsson ES, Abrahamsson H. Health-related quality of life in patients attending a gastroenterology outpatient clinic: functional disorders versus organic diseases. Clin Gastroenterol Hepatol. 2006; 4:187–195. doi: 10.1016/S1542-3565(05)00981-X 16469679 10.1016/s1542-3565(05)00981-x

[5] Den Hollander WJ and Kuipers EJ. Current pharmacotherapy options for gastritis. Expert Opin Pharmacother. 2012; 13(18):2625–2636. doi: 10.1517/14656566.2012.747510 23167300 10.1517/14656566.2012.747510

[6] Liu M, Liu Z. Overview of clinical study on traditional Chinese medicine invigorating spleen and stomach, promoting blood circulation and remove blood stasis in treatment of chronic atrophic gastritis. Zhongguo Zhong Yao Za Zhi. 2012; 37(22):3361–3364. 23373202

[7] Higgins JP, Thompson SG, Deeks JJ, Altman DG. Measuring inconsistency in meta-analyses.BMJ. 2003; 327(7414):557–560. doi: 10.1136/bmj.327.7414.557 12958120 10.1136/bmj.327.7414.557 PMC192859

[8] Xu JW. Huangqi Jianzhong decoction in the treatment of chronic superficial gastritis caused by persistent chronic atrophic gastritis. Shaanxi Journal of Traditional Chinese Medicine. 2016; 37(2):142–144.

[9] Liang MH, Guo LG. Influence of Yiqi Huoxue Yangyin Method on Treatment Effect and Blood Rheology in Chronic Atrophic Gastritis. CHINESE ARCHIVES OF TRADITIONAL CHINESE MEDICINE. 2016;34(7):1704–1707.

[10] Ma XY. Jianpi Tongluo Soup Combined with Western Medicine Treatment of Stomach Deficiency Type of Chronic Atrophic Gastritis and Hp Infection Randomized Controlled Study. JOURNAL OF PRACTICAL TRADITIONAL CHINESE INTERNAL MEDICINE. 2015; 29(11):124–126.

[11] Peng GS, Kuang NF, Guo YJ. Chronic Atrophic Gastritis Parallel Randomized Controlled Study Yiqiyangyin Decoction. JOURNAL OF PRACTICAL TRADITIONAL CHINESE INTERNAL MEDICINE. 2015; 29(2):52–54.

[12] Wang ZX. Thirty-Four Cases of Chronic Atrophy Gastritis Treated with Prescription for Invigorating Spleen and Stomach. HENAN TRADITIONAL CHINESE MEDICINE. 2015; 35(11):2794–2795.

[13] Wang YY. Yiqi Huoxue method for treatment of chronic atrophic gastritis. Journal of Changchun University of Chinese Medicine. 2015; 31(2):361–363.

[14] Zhou JH, Fu ZQ, Deng JP, Li CX, Qiao Z, Zhu WQ, et al Effect of Chinese Drugs for Strengthening Pi, Harmonizing Wei, and Dispersing Blood Stasis on the Expression of Gastric Mucosal Heat Shock Protein 70 in Chronic Atrophic Gastritis Patients. Chinese Journal of Integrated Traditional and Western Medicine. 2015; 35(4):406–410. doi: 10.7661/CJIM.2015.04.0406 26065096

[15] Lu RX, Zhang QP, Hao B. Yiqiyangwei decoction combined with western medicine in the treatment of chronic atrophic gastritis (Report of 60 cases). Medical Research and Education. 2014; 31(4): 30–33, 56.

[16] Zhang CG, Zhang QH, Jia LH. Invigorating Blood Circulation to Weak Suppression Soup Treatment of Chronic Atrophic Gastritis Random Parallel Control Study. JOURNAL OF PRACTICAL TRADITIONAL CHINESE INTERNAL MEDICINE. 2014; 28(5): 56–58.

[17] Li YP. Clinical observation on treating chronic atrophic gastritis in TCM. Clinical Journal of Chinese Medicine. 2014; 6(25):126–127.

[18] Wang JZ, Quan XX. Yiqi Huoxue Yangyin Combined with Western Medicine in the Treatment of Chronic Atrophic Gastritis Randomized and Controlled Study. JOURNAL OF PRACTICAL TRADITIONAL CHINESE INTERNAL MEDICINE. 2013; 27(1):81–82.

[19] Liu DX. Clinical Study of Yiqi Huoxue Huazhuo Jiedu Decoction in the Treatment of Chronic Atrophic Gastritis. CHINA JOURNAL OF CHINESE MEDICINE. 2013; 28(180):745–746.

[20] Chen XB, Zhu N, Li LS, Lu YE, Chen MF. Clinical Observation of Therapy of Integrated Chinese and Western Medicine on Chronic Atrophic Gastritis. SHANXI OF TCM. 2010; 26(9):24–25.

[21] Zheng XY. Chinese herbal medicine new medicine clinical research guiding principle [M]. Beijing: China Medical Science Press 2002:124–129.

[22] The gastroenterology branch of China institute of traditional Chinese medicine. A consensus in traditional Chinese medicine diagnosis and treatment of chronic atrophic gastritis. Journal of Traditional Chinese Medicine. 2010; 51(8):749–753.

[23] Valle J, Kekki M, Sipponen P, Ihamäki T, Siurala M. Long-term course and consequences of Helicobacter pylori gastritis. Results of a 32-year follow-up study. Scand J Gastroenterol. 1996; 31(6):546–550. 8789892 10.3109/00365529609009126

[24] Villako K, Kekki M, Maaroos HI, Sipponen P, Uibo R, Tammur R, et al Chronic gastritis: progression of inflammation and atrophy in a six-year endoscopic follow-up of a random sample of 142 Estonian urban subjects. Scand J Gastroenterol Suppl. 1991; 186:135–141. 1759121 10.3109/00365529109104000

[25] Zabaleta J. Multifactorial etiology of gastric cancer. Methods Mol Biol. 2012; 863:411–435. doi: 10.1007/978-1-61779-612-8_26 22359309 10.1007/978-1-61779-612-8_26 PMC3625139

[26] Centanni M, Marignani M, Gargano L, Corleto VD, Casini A, Delle Fave G, et al Atrophic body gastritis in patients with autoimmune thyroid disease: an underdiagnosed association. Arch Intern Med. 1999; 159(15):1726–1730 10448775 10.1001/archinte.159.15.1726

[27] Neumann WL, Coss E, Rugge M, Genta RM. Autoimmune atrophic gastritis—pathogenesis, pathology and management. Nat Rev Gastroenterol Hepatol. 2013; 10(9):529–541. doi: 10.1038/nrgastro.2013.101 23774773 10.1038/nrgastro.2013.101

[28] Zhang ZH. Review of Pharmacological Study and Clinical Application of *Xiangsha Liujunzi* Decoction. JOURNAL OF LIAONING UNIVERSITY OF TCM. 2013; 15(5):245–247.

[29] Sun WF, Sun GH, Hua PX, Li ZX, Chen JL, Tao WP, et al Effects of Shenxiang Yangwei Power on Precancerous Lesion in Chronic Atrophic Gastritis. Journal of Anhui TCM College. 2001; 20(1):13–15.

[30] Guo YL, Rao J, Pan HF, Fang J. Effect of the Treatment of Jianpi Huayu Jiedu for Patients with Chronic Atrophic Gastritis and its Influence on Cyclin E Protein Expression. Chinese Journal of Experimental Traditional Medical Formulae. 2013; 19(11):292–295.

[31] Lu WM, Shan ZW, Shen H, Wu J, Zhu YH, Zhu CL. Clinical Study of Weishu Capsule in Treating Precancerous Lesions of Chronic Atrophic Gastritis. Chinese Journal of Integrated Traditional and Western Medicine. 1998; 18(12):721–723. 11475717

[32] Matos JI, De Sousa HA, Marcos-Pinto R, Dinis-Ribeiro M. Helicobacter pylori CagA and VacA genotypes and gastric phenotype: a meta-analysis. Eur J Gastroenterol Hepatol. 2013; 25(12):1431–1441. doi: 10.1097/MEG.0b013e328364b53e 23929249 10.1097/MEG.0b013e328364b53e

[33] Sun TT, Wang JL, Fang JY. Quality of RCTs exploring Helicobacter pylori eradication for the prevention of gastric cancer and preneoplastic lesions. Expert Rev Anticancer Ther. 2011; 11(10):1509–1519. doi: 10.1586/era.11.124 21999125 10.1586/era.11.124

[34] Fuccio L, Zagari RM, Eusebi LH, Laterza L, Cennamo V, Ceroni L, et al Meta-analysis: can Helicobacter pylori eradication treatment reduce the risk for gastric cancer? Ann Intern Med. 2009; 151(2):121–128. 19620164 10.7326/0003-4819-151-2-200907210-00009

[35] Xue FB, Xu YY, Wan Y, Pan BR, Ren J, Fan DM. Association of H.pylori infection with gastric carcinoma: a Meta analysis. 2001; 7(6):801–804. 10.3748/wjg.v7.i6.801 PMC4695598 11854905

