# Supplementary material for: The efficacy of Jianpi Yiqi therapy for chronic atrophic gastritis: A systematic review and meta-analysis
Source: PLoS One. 2017 Jul 24;12(7):e0181906. doi: 10.1371/journal.pone.0181906 (PMC5524332; doi:10.1371/journal.pone.0181906)
Supplement: S1 Table — (DOC) [file pone.0181906.s002.doc]

**Table 2. The ingredients of each formula.**

| Author | Ingredients of each formula | | | |
| --- | --- | --- | --- | --- |
| Xu 2016 (8) | *Astragalus membranaceus* (Huang Qi) 15g | *Cynanchum otophyllum* (Bai Shao) 15g | *Polygonatum odoratum* (Yu Zhu) 15g | *Radix Glycyrrhizae preparata* (Zhi Gan Cao) 15g |
|  | *Aconitum carmichaeli Debx* (Fu Zi) 10g | *Amomum villosum Lour* (Sha Ren) 10g | *Hippophae rhamnoides L* (Yi Tang) 10g | *Cinnamomum cassia Presl* (Gui Zhi) 6g |
|  | *Aucklandia lappa Decne* (Mu Xiang) 6g | *Zingiber officinale Rose* (Sheng Jiang) 6g | *Ziziphus jujuba Mill* (Da Zao) 6g |  |
| Liang et al. 2016 (9) | *Astragalus membranaceus* (Huang Qi) 15g | *Codonopsis pilosula (Franch.) Nannf* (Dang Shen) 15g | *Aaugellica sinensis(Oliv) Diels* (Dang Gui) 9g | *Citrus reticulata Blanco* (Chen Pi) 9g |
|  | *Ophiopogon japonicus(Thunb.) Ker-Gawl* (Mai Dong) 15g | *Atractylodes macrocephala Koidz* (Chao Bai Zhu) 9g | *Polygonatum odoratum* (Yu Zhu) 12g | *Glehnia littoralis Fr. Schmidt ex Miq* (Bei Sha Shen) 12g |
|  | *Citrus aurantium L* (Zhi Qiao) 9g | *Citrus medica L.Var. Sarcodactylis Swingle* (Fo Shou) 9g | *Panax notoginseng (Burk.) F. H. Chen* (San Qi) 6g | *Radix Glycyrrhizae preparata* (Zhi Gan Cao) 3g |
|  | *Paeonia lactiflora Pall.* (Chi Shao)12g | *Curcuma wenyujin Y.H.Chen et C.Ling* (Yu Jin)12g |  |  |
| Ma 2015 (10) | *Poria cocos (Schw.) Wol*f (Fu Lin) 20g | *Astragalus membranaceus* (Huang Qi) 20g | *Atractylodes macrocephala Koidz.* (Bai Zhu) 15g | *Salvia miltiorrhiza Bge* (Dan Shen) 20g |
|  | *Codonopsis pilosula (Franch.) Nannf* (Dang Shen) 15g | *Pinellia ternata(Thunb) Breit* (Fa Ban Xia) 9g | *Amomum villosum Lour* (Sha Ren) 6g | *Cinnamomum cassia Presl* (Gui Zhi) 6g |
|  | *Radix Glycyrrhizae preparata* (Zhi Gan Cao) 6g | *Zingiber officinale Rosc* (Gan Jiang) 6g |  |  |
| Peng et al. 2015 (11) | *Salvia miltiorrhiza Bge* (Dan Shen) 10g | *Cynanchum otophyllum* (Bai Shao) 10g | *Ophiopogon japonicus (Thunb.) Ker-Gawl* (Mai Dong) 10g | *Glehnia littoralis Fr. Schmidt ex Miq* (Sha Shen) 10g |
|  | *Aaugellica sinensis(Oliv) Diels* (Dang Gui) 10g | *Pinellia ternata(Thunb) Breit* (Jiang Ban Xia) 9g | *Scutellaria barbataD.Don* (Ban Zhi Lian) 9g | *Radix Glycyrrhizae preparata* (Gan Cao) 9g |
|  | *Citrus aurantium L* (Zhi Qiao) 9g | *Solanum nigrum L* (Long Kui) 9g | *Dolichos lablab L* (Chao Bian Dou) 15g | *Oldenlandia diffusa (willd.) Rox* (Bai Hua She She Cao)15g |
|  | *Codonopsis pilosula (Franch.) Nannf* (Dang Shen) 15g |  |  |  |
| Wang ZX 2015 (12) | *Codonopsis pilosula (Franch.) Nannf* (Dang Shen) 30g | *Astragalus membranaceus* (Huang Qi) 30g | *Poria cocos (Schw.) Wol*f (Fu Lin) 15g | *Atractylodes macrocephala Koidz* (Bai Zhu) 12g |
|  | *Dioscorea opposita Thunb* (Shan Yao) 20g | *Salvia miltiorrhiza Bge* (Dan Shen) 15g | *Rehmannia glutinosa Libosch*  (Sheng Di Huang) 30g | *Aaugellica sinensis(Oliv) Diels* (Dang Gui) 20g |
|  | *Citrus aurantium L* (Zhi Shi) 10g | *Pinellia ternate (Thunb) Breit* (Ban Xia) 10g | *Citrus reticulata Blanco* (Chen Pi) 10g | *Oldenlandia diffusa (willd.) Roxb* (Bai Hua She She Cao) 30g |
|  | *Radix Glycyrrhizae preparata* (Zhi Gan Cao) 6g |  |  |  |
| Wang YY 2015 (13) | *Astragalus membranaceus* (Huang Qi) 30g | *Panax quinquefolium L* (Xi Yang Shen)10g | *Prunus persica (L.)* Batsch (Tao Ren) 6g | *Carthamus tinctorius L* (Hong Hua) 6g |
|  | *Bupleurum chinensis DC*. (Chai Hu) 12g | *Aucklandia lappa Decne* (Mu Xiang) 6g | *Bletilla striata (Thunb.) Reichb.F* (Bai Ji) 9g | *Ligusticum chuanxiong Hort*  (Chuan Xiong) 6g |
|  | *Panax notoginseng (Burk.) F. H.Chen* (Tian San Qi) 9g | *Gallus gallus domesticus Brisson* (Ji Nei Jin) 15g | *Coptis chinensis Franch* (Huang Lian) 3g | *Citrus reticulata Blanco* (Chen Pi) 15g |
| Zhou et al. 2015 (14) | *Astragalus membranaceus* (Huang Qi) 12g | *Corydalis yanhusuo W.T.Wang* (Yan Hu Suo) 10g | *Cyperus rotundus L* (Xiang Fu) 10g | *Crataegus pinnatifida Bge. var. major N.E.Br* (Shan Zha) 12g |
|  | *Cynanchum otophyllum* (Bai Shao) 10g | *Glehnia littoralis Fr. Schmidt ex Miq* (Bei Sha Shen) 10g | *Ophiopogon japonicus (Thunb.) Ker-Gawl* (Mai Dong) 10g | *Salvia miltiorrhiza Bge* (Dan Shen) 12g |
|  | *Radix Glycyrrhizae preparata* (Zhi Gan Cao) 6g |  |  |  |
| Lu et al. 2014 (15) | *Astragalus membranaceus* (Sheng Huang Qi) 20g | *Aaugellica sinensis (Oliv) Diels* (Dang Gui) 15g | *Rehmannia glutinosa Libosch*  (Sheng Di Huang) 15dig | *Taraxacum mongolicum Hand. -Mazz* (Pu Gong Ying) 10g |
|  | *Radix Glycyrrhizae preparata* (Gan Cao) 10g | *Angelica dahurica (Fisch.ex Hoffm.)Benth.et Hook.f.* (Bai zhi) 10g | *Lysimachia christinae Hance* (Jin Qian Cao) 8g | *Lycium chinense Mil1.* (Di Gu Pi) 15g |
|  | *A.kravanh Pierre ex Gagnep.* (Dou Kou) 12g | *Dendrobium loddigesii Rolfe.* (Shi Hu) 18g | *Nelumbo nucifera Gaertn.* (He Geng) 10g |  |
| Zhang et al. 2014 (16) | *Codonopsis pilosula (Franch.) Nannf* (Dang Shen) 20g | *Atractylodes lancea* ( *Thunb.*) *DC* (Cang Zhu) 10g | *Atractylodes macrocephala Koidz* (Bai Zhu) 10g | *Poria cocos (Schw.) Wol*f (Fu Lin) 15g |
|  | *Radix Glycyrrhizae preparata* (Zhi Gan Cao) 10g | *Curcuma phaeocaulis Val* (E Zhu) 10g | *Salvia miltiorrhiza Bge* (Dan Shen) 15g | *Pinellia ternate (Thunb) Breit* (Jiang Ban Xia) 10g |
|  | *Citrus reticulata Blanco* (Chen Pi) 6g |  |  |  |
| Li 2014 (17) | *Ophiopogon japonicus (Thunb.) Ker-Gawl* (Mai Dong) 8g | *Glehnia littoralis Fr. Schmidt ex Miq* (Sha Shen) 12g | *Aaugellica sinensis (Oliv) Diels* (Dang Gui) 10g | *Scutellaria barbataD.Don* (Ban Zhi Lian) 8g |
|  | *Coptis chinensis Franch.* (Huang Lian)6g | *Dolichos lablab L* (Chao Bian Dou) 12g | *Cynanchum otophyllum* (Bai Shao) 12g | *Pinellia ternate (Thunb) Breit* (Jiang Ban Xia) 8g |
|  | *Radix Glycyrrhizae preparata* (Gan Cao) 8g | *Oldenlandia diffusa (willd.) Roxb* (Bai Hua She She Cao) 14g |  |  |
| Wang et al. 2013 (18) | *Cynanchum otophyllum* (Bai Shao) 10g | *Salvia miltiorrhiza Bge* (Dan Shen) 10g | *Glehnia littoralis Fr. Schmidt ex Miq* (Sha Shen) 10g | *Ophiopogon japonicus (Thunb.) Ker-Gawl* (Mai Dong) 10g |
|  | *Aaugellica sinensis (Oliv) Diels* (Dang Gui) 10g | *Scutellaria barbataD. Don* (Ban Zhi Lian) 9g | *Pinellia ternate (Thunb) Breit* (Jiang Ban Xia) 9g | *Solanum nigrum L* (Long Kui) 9g |
|  | *Radix Glycyrrhizae preparata* (Gan Cao) 9g | *Citrus aurantium L* (Zhi Qiao) 9g | *Codonopsis pilosula (Franch.) Nannf* (Dang Shen) 15g | *Dolichos lablab L* (Chao Bian Dou) 15g |
|  | *Oldenlandia diffusa (willd.) Roxb* (Bai Hua She She Cao) 15g |  |  |  |
| Liu 2013 (19) | *Astragalus membranaceus* (Huang Qi) 30g | *Codonopsis pilosula (Franch.) Nannf* (Dang Shen) 30g | *Atractylodes macrocephala Koidz* (Chao Bai Zhu) 10g | *Poria cocos (Schw.)Wol*f (Fu Lin) 10g, |
|  | *Citrus reticulata Blanco* (Chen Pi) 10g, | *Citrus aurantium L* (Zhi Shi) 10g | *Taraxacum mongolicum Hand. -Mazz* (Pu Gong Ying) 30g | *Oldenlandia diffusa (willd.) Roxb* (Bai Hua She She Cao) 30g |
|  | *Aaugellica sinensis(Oliv) Diels* (Dang Gui) 10g | *Bletilla striata (Thunb.) Reichb. F.* (Bai Ji) 30g | *Corydalis yanhusuo W.T.Wang* (Yan Hu Suo) 10g | *Pinellia ternata(Thunb) Breit.* (Ban Xia) 10g |
|  | *Coix lacryma-jobi L.var.ma-yuen (Roman.) Stapf* (Yi Yi Ren) 30g | *Gallus gallus domesticus Brisson* (Ji Nei Jin) 10g | *Dioscorea opposita Thunb*  (Shan Yao) 25g | *Aucklandia lappa Decne* (Mu Xiang) 10g |
|  | *Scutellaria barbataD.Don* (Ban Zhi Lian) 10g |  |  |  |
| Chen et al. 2010 (20) | *Codonopsis pilosula (Franch.) Nannf* (Dang Shen) 20g | *Dioscorea opposita Thunb*. (Shan Yao) 20g | *Astragalus membranaceus* (Zhi Huang Qi) 15g | *Poria cocos (Schw.) Wol*f (Fu Lin) 15g |
|  | *Atractylodes macrocephala Koidz.* (Chao Bai Zhu) 10g | *Coptis chinensis Franch.* (Huang Lian)6g | *Aaugellica sinensis(Oliv) Diels* (Dang Gui) 10g | *Citrus medica L.Var. Sarcodactylis Swingle* (Fo Shou) 10g |
|  | *Amomum villosum Lour* (Sha Ren) 6g | *Glehnia littoralis Fr. Schmidt ex Miq* (Sha Shen) 6g | *Evodia rutaecarpa (Juss.) Benth.* (Wu Zhu Yu) 5g | *Radix Glycyrrhizae preparata* (Zhi Gan Cao) 5g |
|  | *Panax notoginseng (Burk.) F. H. Chen* (San Qi Fen) 3g |  |  |  |
