# Supplementary material for: The efficacy of Jianpi Yiqi therapy for chronic atrophic gastritis: A systematic review and meta-analysis
Source: PLoS One. 2017 Jul 24;12(7):e0181906. doi: 10.1371/journal.pone.0181906 (PMC5524332; doi:10.1371/journal.pone.0181906)
Supplement: S2 Table — (DOC) [file pone.0181906.s003.doc]

**Table 3. Frequencies of usage and distribution in TCM.**

| Chinese herbs | Frequency | Rate(%) | Chinese herbs | Frequency | Rate(%) |
| --- | --- | --- | --- | --- | --- |
| *Radix Glycyrrhizae preparata*(Gan Cao) | 11 | 7.1 | *Cinnamomum cassia Presl*(Gui Zhi) | 2 | 1.3 |
| *Astragalus membranaceus*(Huang Qi) | 9 | 5.8 | *Corydalis yanhusuo W.T.Wang* (Yan Hu Suo) | 2 | 1.3 |
| *Codonopsis pilosula (Franch.)Nannf.*(Dang Shen) | 8 | 5.2 | *Bletilla striata (Thunb.) Reichb. F.*(Bai Ji) | 2 | 1.3 |
| *Aaugellica sinensis(Oliv) Diels.*(Dang Gui) | 8 | 5.2 | *Lycium chinense Mil1.*(Di Gu Pi) | 1 | 0.6 |
| *Oldenlandia diffusa (willd.) Roxb.*(Bai Hua She She Cao) | 6 | 3.8 | *Evodia rutaecarpa (Juss.) Benth.*(Wu Zhu Yu) | 1 | 0.6 |
| *Salvia miltiorrhiza Bge*.(Dan Shen) | 6 | 3.8 | *Lysimachia christinae Hance*(Jin Qian Cao) | 1 | 0.6 |
| *Pinellia ternata(Thunb) Breit.*(Ban Xia) | 6 | 3.8 | *Coix lacryma-jobi L.var.ma-yuen (Roman.) Stapf*(Yi Yi Ren) | 1 | 0.6 |
| *Glehnia littoralis Fr. Schmidt ex Miq.*(Sha Shen) | 6 | 3.8 | *Ligusticum chuanxiong Hort*.(Chuan Xiong) | 1 | 0.6 |
| *Atractylodes macrocephala Koidz.*(Bai Zhu) | 6 | 3.8 | *Bupleurum chinensis DC*.(Chai Hu) | 1 | 0.6 |
| *Cynanchum otophyllum*(Bai Shao) | 5 | 3.2 | *Carthamus tinctorius L.*(Hong Hua) | 1 | 0.6 |
| *Poria cocos (Schw.)Wol*f(Fu Lin) | 5 | 3.2 | *Prunus persica(L.)*Batsch(Tao Ren) | 1 | 0.6 |
| *Ophiopogon japonicus(Thunb.)Ker-Gawl.*(Mai Dong) | 5 | 3.2 | *Panax quinquefolium L.*(Xi Yang Shen) | 1 | 0.6 |
| *Citrus reticulata Blanco*(Chen Pi) | 5 | 3.2 | *Dendrobium loddigesii Rolfe.*(Shi Hu) | 1 | 0.6 |
| *Scutellaria barbataD.Don.*(Ban Zhi Lian) | 4 | 2.6 | *Zingiber officinale Rosc*.(Gan Jiang) | 1 | 0.6 |
| *Dolichos lablab L*.(Bian Dou) | 3 | 1.9 | *Curcuma phaeocaulis Val.*(E Zhu) | 1 | 0.6 |
| *Coptis chinensis Franch.*(Huang Lian) | 3 | 1.9 | *Curcuma wenyujin Y.H.Chen et C.Ling*(Yu Jin) | 1 | 0.6 |
| *Dioscorea opposita Thunb*.(Shan Yao) | 3 | 1.9 | *Atractylodes lancea* ( *Thunb.*) *DC.*(Cang Zhu) | 1 | 0.6 |
| *Amomum villosum Lour*(Sha Ren) | 3 | 1.9 | *Aconitum carmichaeli Debx*(Fu Zi) | 1 | 0.6 |
| *Aucklandia lappa Decne*(Mu Xiang) | 3 | 1.9 | *Hippophae rhamnoides L*(Yi Tang) | 1 | 0.6 |
| *Citrus aurantium L.*(Zhi Qiao) | 3 | 1.9 | *Zingiber officinale Rose*(Sheng Jiang) | 1 | 0.6 |
| *Panax notoginseng (Burk.) F. H. Chen*(San Qi) | 3 | 1.9 | *Ziziphus jujuba Mill*(Da Zao) | 1 | 0.6 |
| *Gallus gallus domesticus Brisson*(Ji Nei Jin) | 2 | 1.3 | *Cyperus rotundus L.*(Xiang Fu) | 1 | 0.6 |
| *Solanum nigrum L.*(Long Kui) | 2 | 1.3 | *Crataegus pinnatifida Bge.var.major N.E.Br.*(Shan Zha) | 1 | 0.6 |
| *Citrus aurantium L.*(Zhi Shi) | 2 | 1.3 | *Paeonia lactiflora Pall.*(Chi Shao) | 1 | 0.6 |
| *Polygonatum odoratum* (Yu Zhu) | 2 | 1.3 | *A.kravanh Pierre ex Gagnep.*(Dou Kou) | 1 | 0.6 |
| *Rehmannia glutinosa Libosch*.(Sheng Di Huang) | 2 | 1.3 | *Angelica dahurica (Fisch.ex Hoffm.)Benth.et Hook.f.*(Bai zhi) | 1 | 0.6 |
| *Citrus medica L. Var. Sarcodactylis Swingle*(Fo Shou) | 2 | 1.3 | *Nelumbo nucifera Gaertn.*(He Geng) | 1 | 0.6 |
| *Taraxacum mongolicum Hand.-Mazz*.(Pu Gong Ying) | 2 | 1.3 |  |  |  |
