# Supplementary material for: The efficacy of Jianpi Yiqi therapy for chronic atrophic gastritis: A systematic review and meta-analysis
Source: PLoS One. 2017 Jul 24;12(7):e0181906. doi: 10.1371/journal.pone.0181906 (PMC5524332; doi:10.1371/journal.pone.0181906)
Supplement: S3 Table — (DOC) [file pone.0181906.s004.doc]

**Table 4. Chinese herbs classification.**

| TCM Category | Chinese herbs | | | |
| --- | --- | --- | --- | --- |
| Invigorating spleen  and reinforcing qi  (Jianpi Yiqi) | *Radix Glycyrrhizae preparata* (Gan Cao) | *Codonopsis pilosula (Franch.)Nannf.* (Dang Shen) | *Astragalus membranaceus* (Huang Qi) | *Ziziphus jujuba Mill* (Da Zao) |
|  | *Atractylodes macrocephala Koidz* (Bai Zhu) | *Dolichos lablab L* (Bian Dou) | *Dioscorea opposita Thunb* (Shan Yao) | *Panax quinquefolium L* (Xi Yang Shen) |
|  | *Atractylodes lancea* (*Thunb*) *DC* (Cang Zhu) | *Hippophae rhamnoides L* (Yi Tang) |  |  |
| Regulating qi  (Li qi) | *Citrus reticulata Blanco* (Chen Pi) | *Citrus aurantium L* (Zhi Qiao) | *Aucklandia lappa Decne* (Mu Xiang) | *Citrus aurantium L* (Zhi Shi) |
|  | *Cyperus rotundus L* (Xiang Fu) | *Amomum villosum Lour* (Sha Ren) | *Citrus medica L.Var. Sarcodactylis Swingle* (Fo Shou) | *Corydalis yanhusuo W.T.Wang* (Yan Hu Suo) |
|  | *Cinnamomum cassia Presl* (Gui Zhi) | *Curcuma wenyujin Y.H.Chen et C.Ling* (Yu Jin) | *Nelumbo nucifera Gaertn.* (He Geng) |  |
| Relieving the depressed liver (Shugan Jieyu) | *Bupleurum chinensis DC* (Chai Hu) | *Cyperus rotundus L* (Xiang Fu) | *Citrus medica L.Var. Sarcodactylis Swingle* (Fo Shou) |  |
| Promoting digestion  and relieving stasis  (Xiaoshi Huaji) | *Crataegus pinnatifida Bge.var.major N.E.Br.* (Shan Zha) | *Gallus gallus domesticus Brisson* (Ji Nei Jin) |  |  |
| Blood activiating  and stasis dissolving  (Huoxue Huayu) | *Ligusticum chuanxiong Hort* (Chuan Xiong) | *Salvia miltiorrhiza Bge* (Dan Shen) | *Curcuma phaeocaulis Val* (E Zhu) | *Aaugellica sinensis(Oliv) Diels* (Dang Gui) |
|  | *Panax notoginseng (Burk.) F. H. Chen* (San Qi) | *Bletilla striata (Thunb.) Reichb.F* (Bai Ji) | *Carthamus tinctorius L* (Hong Hua) | *Prunus persica(L.)* Batsch (Tao Ren) |
|  | *Curcuma wenyujin Y.H.Chen et C.Ling* (Yu Jin) | *Paeonia lactiflora Pall.* (Chi Shao) |  |  |
| Resolving dampness  (Chu shi) | *Pinellia ternata(Thunb) Breit* (Ban Xia) | *Poria cocos (Schw.)Wol*f (Fu Lin) | *Atractylodes lancea* ( *Thunb.*) *DC* (Cang Zhu) | *Coix lacryma-jobi L.var.ma-yuen (Roman.) Stapf* (Yi Yi Ren) |
|  | *A.kravanh Pierre ex Gagnep.* (Dou Kou) |  |  |  |
| Clearing away heat  (Qing re) | *Coptis chinensis Franch* (Huang Lian) | *Taraxacum mongolicum Hand.-Mazz* (Pu Gong Ying) | *Oldenlandia diffusa (willd.) Roxb.* (Bai Hua She She Cao) | *Scutellaria barbataD.Don* (Ban Zhi Lian) |
|  | *Rehmannia glutinosa Libosch* (Sheng Di Huang) | *Solanum nigrum L.* (Long Kui) | *Lycium chinense Mil1.* (Di Gu Pi) | *Lysimachia christinae Hance* (Jin Qian Cao) |
| Warming middle-jiao to dispel cold  (Wenzhong Sanhan) | *Evodia rutaecarpa (Juss.) Benth.* (Wu Zhu Yu) | *Zingiber officinale Rose* (Sheng Jiang) | *Zingiber officinale Rosc*. (Gan Jiang) | *Aconitum carmichaeli Debx* (Fu Zi) |
|  | *Angelica dahurica (Fisch.ex Hoffm.) Benth.et Hook.f.* (Bai zhi) |  |  |  |
| Nourishing Yin  (Zi yin) | *Cynanchum otophyllum* (Bai Shao) | *Glehnia littoralis Fr. Schmidt ex Miq.* (Sha Shen) | *Ophiopogon japonicus (Thunb.)Ker-Gawl.* (Mai Dong) | *Panax quinquefolium L* (Xi Yang Shen) |
|  | *Rehmannia glutinosa Libosch*. (Sheng Di Huang) | *Polygonatum odoratum* (Yu Zhu) | *Dendrobium loddigesii Rolfe.* (Shi Hu) |  |
