# Supplementary material for: The efficacy of Jianpi Yiqi therapy for chronic atrophic gastritis: A systematic review and meta-analysis
Source: PLoS One. 2017 Jul 24;12(7):e0181906. doi: 10.1371/journal.pone.0181906 (PMC5524332; doi:10.1371/journal.pone.0181906)
Supplement: S4 Table — (DOC) [file pone.0181906.s005.doc]

**Table 6. GRADE quality grading evaluation.**

| Quality assessment | | | | | | | No of patients | | Effect | | Quality | Importance |
| --- | --- | --- | --- | --- | --- | --- | --- | --- | --- | --- | --- | --- |
|  |  |  |  |  |  |  |  |  |  |  |  |  |
| No of studies | Design | Risk of bias | Inconsistency | Indirectness | Imprecision | Other considerations | Jianpi Yiqi Therapy or combined with conventional western medicine | Conventional western medicine | Relative (95% CI) | Absolute |  |  |
| Effective rate (Jianpi Yiqi Therapy Vesus. conventional western medicine) | | | | | | | | | | | | |
| 4 | randomised trials | very serious^1,2^ | serious^3^ | serious^4^ | very serious^5^ | none^6^ | 179/189 (94.7%) | 119/182 (65.4%) | RR 1.41 (1.27 to 1.57) | 268 more per 1000 (from 177 more to 373 more) | ÅOOOVERY LOW | CRITICAL |
|  |  |  |  |  |  |  |  | 70.2% |  | 288 more per 1000 (from 190 more to 400 more) |  |  |
| Effective rate (Jianpi Yiqi Therapy combined with conventional western medicine Versus. conventional western medicine) | | | | | | | | | | | | |
| 7 | randomised trials | very serious^1,2^ | serious^3^ | serious^4^ | very serious^5^ | none^6^ | 246/265 (92.8%) | 189/263 (71.9%) | RR 1.27 (1.17 to 1.38) | 194 more per 1000 (from 122 more to 273 more) | ÅOOOVERY LOW | CRITICAL |
|  |  |  |  |  |  |  |  | 72% |  | 194 more per 1000 (from 122 more to 274 more) |  |  |
| Subgroup analysis (different treatment courses of 4, 8, 12 weeks among the included studies) | | | | | | | | | | | | |
| 11 | randomised trials | very serious^1,2^ | serious^3^ | serious^4^ | very serious^5^ | none^6^ | 425/454 (93.6%) | 308/445 (69.2%) | RR 1.32 (1.24 to 1.41) | 221 more per 1000 (from 166 more to 284 more) | ÅOOOVERY LOW | CRITICAL |
|  |  |  |  |  |  |  |  | 72% |  | 230 more per 1000 (from 173 more to 295 more) |  |  |

Annotation:

1 No double blinding

2 No allocation concealment

3 Less follow-up
4 Discrepancies in interventions

5 Small simple sizes

6 No explanation was provided
