# Supplementary material for: The efficacy of Jianpi Yiqi therapy for chronic atrophic gastritis: A systematic review and meta-analysis
Source: PLoS One. 2017 Jul 24;12(7):e0181906. doi: 10.1371/journal.pone.0181906 (PMC5524332; doi:10.1371/journal.pone.0181906)
Supplement: S1 File — (DOC) [file pone.0181906.s006.doc]

We comprehensively searched for publications in PubMed database from its inception through November 1, 2016: (“chronic atrophic gastritis OR atrophic gastritis OR precancerous lesions of gastric cancer”) AND (“traditional Chinese medicine OR Chinese herbal medicine OR herbal formula OR herbs OR alternative medicine OR Jianpi OR Yiqi”) AND (“randomized controlled trial”).
